# Evaluation of innovative dual-layer modified polyethersulfone membranes in the control of biofouling

**DOI:** 10.1038/s41598-026-48923-3

**Published:** 2026-05-07

**Authors:** Nermine Nasser, Mohamed Salah El-Din Hassouna, Noha Salem, Ranya Amer, Sherif H. Kandil, Norhan Nady

**Affiliations:** 1 Department of Environmental Studies, Institute of Graduate Studies and Research, Alexandria University, Alexandria, Egypt; 2 Department of Environmental Biotechnology, Genetic Engineering and Biotechnology Research Institute (GEBRI), City of Scientific Research and Technological Applications (SRTA-City), New Borg El-Arab City, Egypt; 3 Polymeric Materials Research Department, Advanced Technologies and New Materials Research Institute (ATNMRI), City of Scientific Research and Technological Applications (SRTA-City), New Borg El-Arab City, Egypt; 4 Faculty of Biotechnology, October University for Modern Sciences and Arts (MSA University), Giza, Egypt; 5 Department of Materials Science, Institute of Graduate Studies and Research, Alexandria University, Alexandria, Egypt

**Keywords:** Aminophenol, Dual-layered modification, Laccase, Membrane biofouling, Phenolic acids, Polyethersulfone, Biochemistry, Biotechnology, Chemistry, Environmental sciences, Materials science, Microbiology

## Abstract

This study uniquely demonstrates the innovative combination of aminophenol and phenolic acids through laccase-catalyzed processes on polyethersulfone (PES) surfaces. Firstly, PES membranes were modified via laccase-catalyzed polymerization of 3-aminophenol (3-AP), then a second layer with either 4-hydroxybenzoic acid (B), gallic acid (G), syringic acid (S), or vanillic acid (V) was integrated using the same laccase-catalyzed polymerization method. The B/3-AP/PES and S/3-AP/PES membranes (using 4-hydroxybenzoic acid and syringic acid as the second modification layer) had better hydrophilicity as the contact angle was reduced from 44.1° (one-layered 3-AP/PES) to 23.8° and 27.9°, respectively, alongside significant increases in the root-mean-square (RMS) roughness (59 nm for unmodified PES vs. 180.2 and 385 nm for B/3-AP/PES and S/3-AP/PES, respectively). Atomic Force Microscopy (AFM) imaging revealed brush-like architectures for 3-AP/PES and B/3-AP/PES, while it was pancake-like in S/3-AP/PES. MIC testing showed that bacterial inhibition could reach 99.9%. Microbial evaluations of biofilm formation showed that B/3-AP/PES gave the highest reduction in the detached bacterial count (77%); this was concomitant with lower hemocytometer cell counts. Scanning Electron Microscopy (SEM) confirmed the reduction of bacterial adhesion. This study introduces a new approach of enzymatically grafting aminophenol layer as a stable anchoring platform for dual-layered modification by natural phenolic compounds.

## Introduction

As the demand for water rises, desalination technology emerges as a feasible solution to water scarcity challenges. Reverse osmosis (RO) is one of the most renowned membrane-based desalination techniques, particularly in the Mediterranean region^1^. The major obstacle in the membrane-based desalination is the membrane fouling, which occurs when substances accumulate on the membrane surface or within its pores, leading to decreased performance and material degradation^2^. It can involve various types, including inorganic scaling, organic fouling, and biofouling, which is contributing to over 45% of all fouling issues. Biofouling is a significant challenge in nanofiltration (NF) and RO systems and results from bacterial growth that forms a thick biofilm, clogging the membrane^1^. The impacts of biofouling include reduced membrane flux, increased pressure differentials, membrane degradation, higher salt passage, and greater energy consumption^3^. Various techniques have been employed to manage and reduce membrane biofouling. A common approach involves continuously dosing feed water with biocides or antimicrobial agents, followed by high-velocity detergent cleaning to remove organic debris. Other methods include inhibiting quorum sensing (QS) using electrical fields to reduce fouling during ultrafiltration. QS molecules facilitate bacterial signaling and communication^4^. Silver compounds and silver ions (Ag^+^) have also proven effective in controlling microbial growth. The fouling process begins with microorganisms attaching to the membrane surface by the extracellular polymeric substances (EPS), which are rich in organic compounds like polysaccharides and proteins. Preventing this initial attachment is likely more effective than targeting already-attached microorganisms, which can help control biofouling and reduce operational costs in desalination systems^3^.

Polyethersulfone (PES) membranes are extensively utilized in desalination and industrial applications for separation and purification due to their chemical and thermal stability, as well as their excellent oxidative resistance^5^. However, PES membranes are prone to fouling, which can lead to a drastic reduction in flux as they adsorb pollutants such as proteins and microorganisms either on their outer surface or within their pores due to the inherent hydrophobic characteristics of PES. Surface modification of PES membranes is one strategy to reduce fouling^6^. Factors such as the hydrophilicity and structure of the membrane’s surface play a crucial role in repelling proteins and enhancing the antifouling performance of membranes without compromising their mechanical and thermal properties. Various methods exist for creating hydrophilic surfaces, including coating, blending, composite, chemical, grafting, or combinations of these techniques^7^. Grafting polymer brushes onto membrane surfaces can significantly reduce foulant attachment through steric hindrance and osmotic effects^8^.

**Fig. 1:**
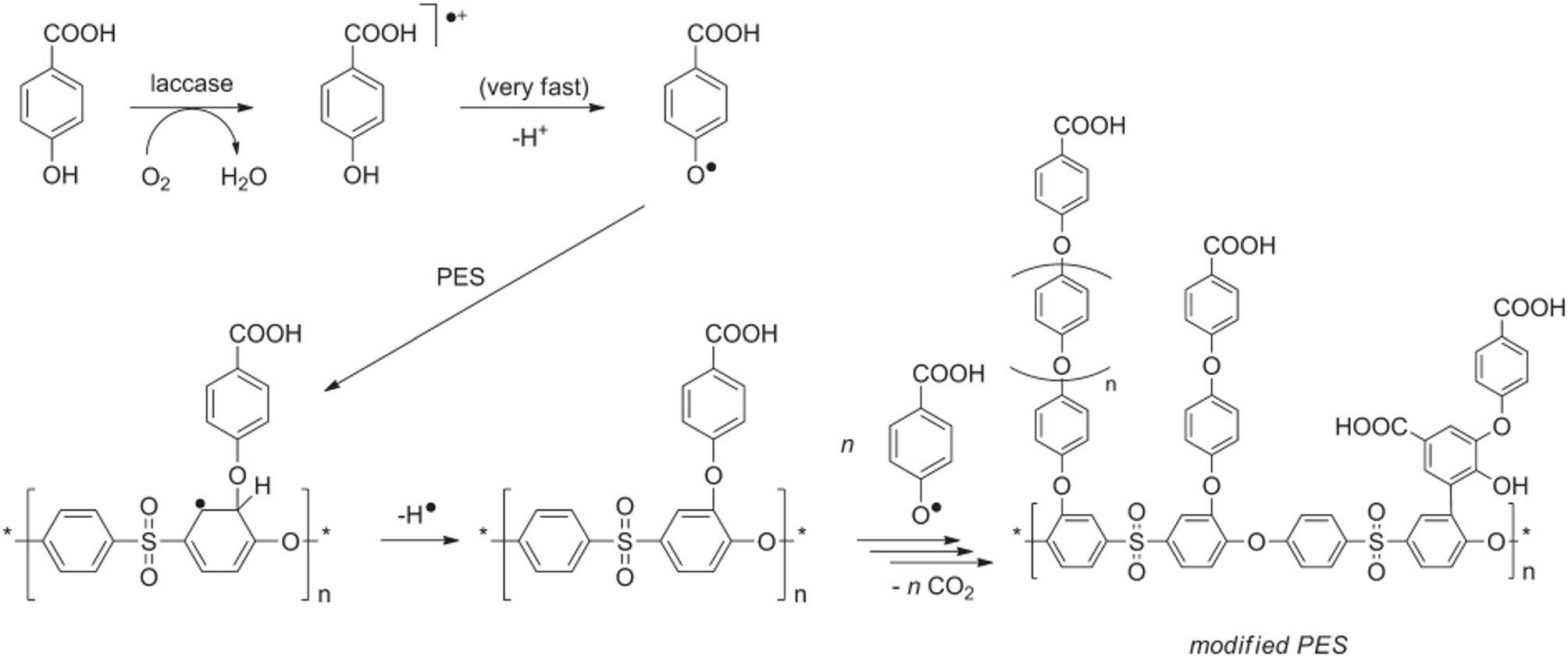
Proposed mechanism for the generation of reactive 4-hydroxybenzoic acid radicals via laccase, along with the subsequent grafting of these radicals onto polyethersulfone (PES) membranes^12^.

**Fig. 2:**
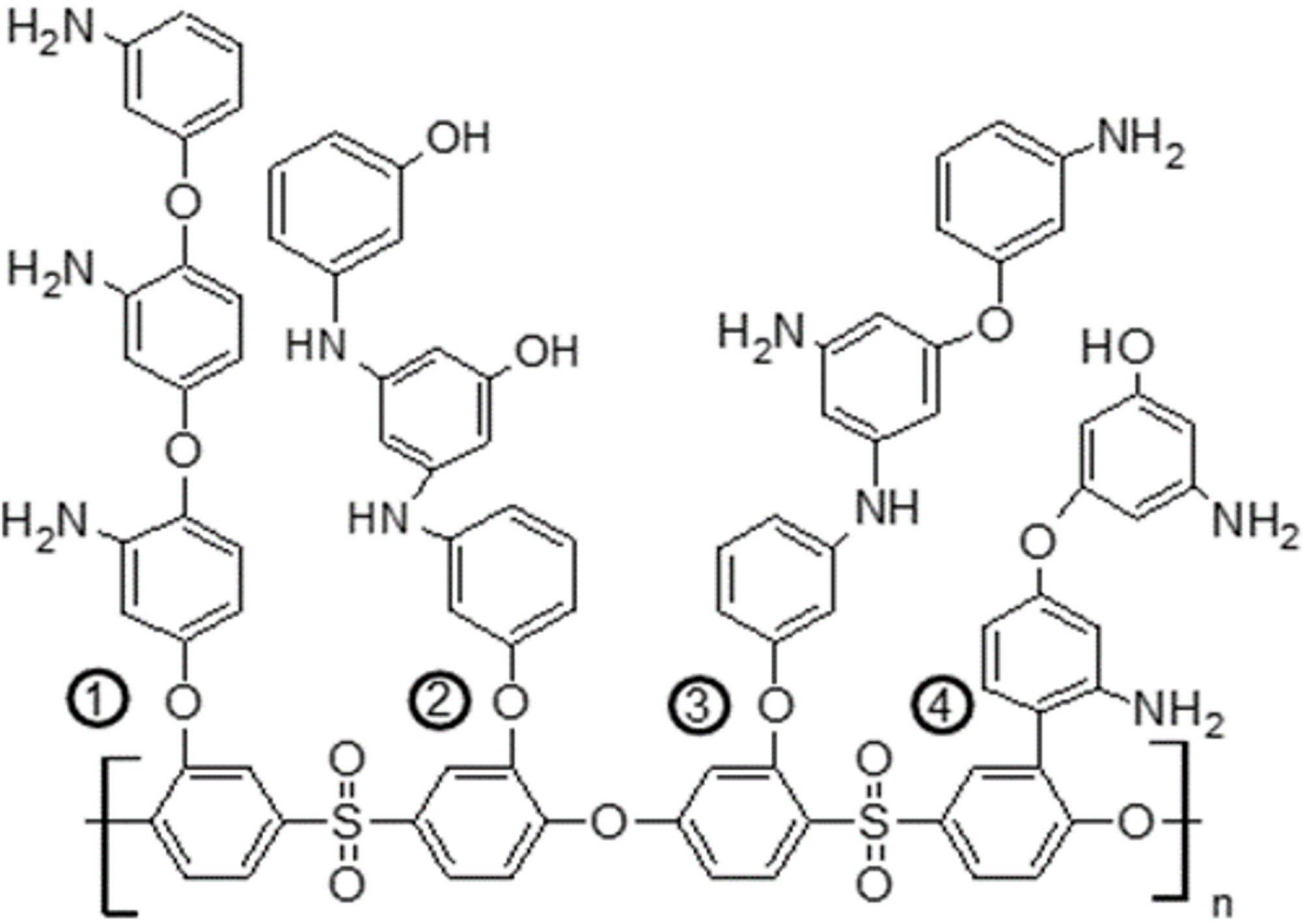
The proposed modifier layers created on the surface of polyethersulfone (PES) membranes using 3-aminophenol^8^.

One innovative approach involves using laccase enzyme from the fungus *Trametes versicolor* to generate free radicals and facilitate covalent bonding to the PES membrane through bio-grafting^7^, as shown in Fig. 1. This enzymatic grafting method offers health and safety benefits by eliminating the need for reactive chemicals. Laccase acts as a biocatalyst to create antifouling layer that reduce protein adsorption, effectively suppressing biofilm formation and biofouling. Aminophenols (APs) are important electrochemical materials that share characteristics with both anilines and phenols due to their oxidizable functional groups –NH_2_ and –OH. APs were chosen as substrates for laccase enzyme in eco-friendly polymerization to enhance PES membranes^9^. The best protein repellence occurs with low modifier concentrations; for example, 5 mM 4-AP that was modified for 120 min at 25 °C achieved a 90.8% reduction in protein adsorption^10^. However, at high monomer concentrations, polymer chains can crosslink or interfere with each other during long modification times^9^. Membranes modified with 3-AP showed improved permeability as the amino groups enhance hydrophilicity^11^, the proposed modifier layers created on the surface of polyethersulfone (PES) membranes using 3-aminophenol shown in Fig. 2.

Operational factors such as temperature, pH and salinity affect the performance and antifouling behavior of the modified membranes. Seawater temperature is the most important physicochemical parameter of coastal surface waters. Increasing temperature results in enhanced mass transfer, and a higher permeate flux, which decrease the affinity of particles deposition and accumulation^13^. At 15, 25 and 35 °C tested temperatures, the colloids average size were 200, 80 and 70 nm, respectively; the larger size of organic foulant at 15 °C resulted in a cake layer deposition and bigger flux decline^14^. On the other hand, many researchers confirmed that fouling conditions were directly dependent on pH and also ionic strength as the result of a balance between electrostatic, solubility and hydrophobic effects, which were related to protein–protein and protein–membrane interactions^15,16^. The pH degree enables the solution to possess charges that can cause the repulsion or attraction of the particles towards the membrane. When membranes are charged negatively by functional groups like carboxylic and sulfonic acid, on the other hand, positively charged membranes can originate from amino groups that accept hydrogen ions in solutions of acidic pH^13^. Another study emphasized that the acidic medium was more preferred to membrane fouling and deterioration; fouling potential increases with increasing acidity of the feed solution and a relatively sticky and strongly attached deposit accumulated^17^. The filtration resistance of the original membrane and pore blockage decreased slightly with an increase in pH, and mitigating membrane fouling by decreasing the cake layer significantly. It was recorded that the membrane flux decreased in the acidic environment^18^. It was also reported that wastewater with high salinity may significantly change foulant properties and subsequent fouling propensity during ultrafiltration^19^. Higher salinity levels promoted cake layer formation, potentially, stimulating QS activity and seriously weakened membrane rejection^20^. In another research, the reduction in permeate flux of RO membranes and total dissolved solid (TDS) rejection were evidenced at low salinity, attributed to marked cell multiplication and release of extracellular polymeric substances, whilst a relatively stable flux was observed at medium and high salinity^21^. A decrease in rejection of inorganic solute by the same fouled membranes is due to the combined effect of reduced surface charge and thickness of the top selective layer of membranes^17^.

Plant phenolic compounds gained more attention due to their potential as effective antimicrobial agents. This interest stems mostly from the fact that using natural antimicrobials does not cause bacterial resistance to evolve^4^. The antimicrobial activity of phenolic compounds can be represented in many different ways such as affecting the permeability of both cell walls and cell membranes, releasing the intracellular constituents, interfering with membrane functions like electron transport, nutrient uptake, protein, nucleic acid synthesis and enzyme activity^22^. Hydrobenzoic acids include commonly; 4-hydroxybenzoic (B), gallic (G), vanillic (V), and syringic (S) acids are subgroup of phenolic acids. The main potential of phenolic compounds as antibacterial agents they have the ability to imitate and confuse QS signals of bacteria^4^. For example, increasing of G concentration clearly reduced the glucose uptake of *E. coli* in the medium and damaged the outer and inner bacterial membranes, and suppressed mRNA expressions which is involved in membrane permeability^23^. The determination of viable bacteria in the biofilm revealed that G penetrated the biofilm to kill *S. aureus*, thus the polysaccharide slime formation was reduced^24^. In addition, gallic, 4-hydroxybenzoic, syringic, and vanillic acids could inhibit bacterial growth and ethanol production in *E. coli* by collapsing ion gradients and increasing internal anion concentrations^25^.

As grafting is a promising method to enhance the wettability and improve antifouling properties by introducing hydrophilic molecules onto the membrane surface^26^, there is the layer-by-layer (LBL) modification approach which can decrease fouling problems made by microorganisms or by organic compounds such as proteins. With the extended multi-step treatment for membranes to induce LBL grafting, the contact angle (CA) potentially decreases and dual effect of antimicrobial and anti-attachment can be achieved. In case of polyethylenimine polymers LBL grafting on Polyethylene terephthalate membrane, CA of less than 25° can be obtained, which outperforms most of the reported results of grafting modifications^26^. It was reported that a membrane which has hydrophilic layer on one side and another side is a hydrophobic layer, that membrane can inhibit most of foulants. The hydrophilic layer repels the hydrophobic compounds and prevents their adhesion on the surface, and the hydrophobic layer mitigates the wetting problem for most membranes^27^. In addition, dual-layer (DL) membranes using two different materials as two layers to possess maximum performance for diverse polymeric pairs^28^. Yin *et al.* demonstrated the dual-layer asymmetric polymer support fabrication of the ZnO-PES/PES^29^.

**Fig. 3:**
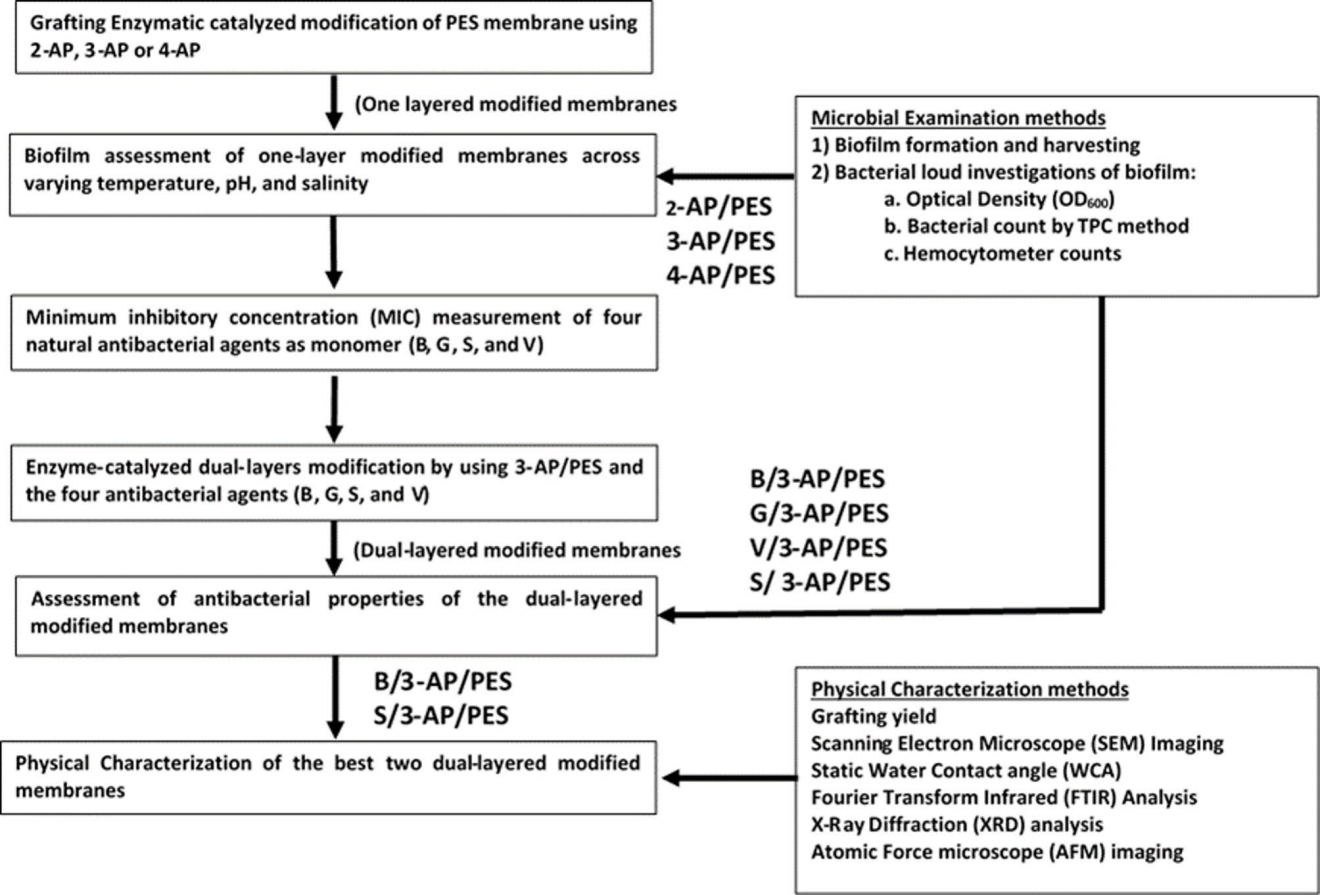
Flowchart for membrane modification and characterization.

The aim of this work is to study the antibacterial and antibiofouling efficiency of the novel green modified PES membrane under simulated environmental seawater conditions. While the previously obtained low-fouling anti-attachment modified aminophenol-PES surface was chosen to add another layer of antibacterial agents such as B, G, V, and S as antimicrobial second layer. With respect to the rationale for the layer sequence, the laccase enzyme is employed to generate free radicals, thereby facilitating covalent bonding through a bio-grafting process in two distinct stages. In this context, the enzyme acts solely as a biocatalyst for the polymerization reaction. The primary function of the 3-aminophenol base layer is to maintain a spatial distance between bacterial cells and the membrane surface. Concurrently, the second layer comprised of phenolic acids serves as an antibacterial agent and it is designed to remain in direct contact with the bacterial cell surface. The anti-attachment properties of the 3-aminophenol layer ensure that both viable and non-viable cells are effectively kept away from the membrane surface. This layered strategy significantly reduces bacterial adhesion while allowing the antibacterial agent to exert its effects, thereby enhancing the overall efficacy of the membrane system. No prior research has successfully combined aminophenol and phenolic acids through laccase catalysis on polyethersulfone (PES) surfaces, according to the authors’ knowledge. Thus the main aim of this research is to study the anti-attachment and anti-microbial efficiency of dual-layer modified membranes on resist biofouling. This would lead to low membrane replacement costs as well as protect the receiving water bodies from contaminants in the reject water. Figure 3.

## Materials and methods

### Materials

2-aminophenol (2-AP, 99.5%), 3-aminophenol (3-AP, 99.8%), 4-aminophenol (4-AP, 99%), Laccase (Sigma-Aldrich) from *Trametes versicolor* (> 0.5 U/ml), acetic acid (99%), sodium acetate (anhydrous, 99%), dichloromethane (DCM, 99.9%) and glucose were obtained from Sigma-Aldrich. Sodium phosphate monobasic and disodium hydrogen phosphate 2- 2-hydrate (extra pure). Ethanol (Analytical reagent grade), Phosphoric acid 85% and Sulfuric acid were obtained from Fisher. Sodium chloride (NaCl) was obtained from MP BioMrdicals. Vanillic acid (98%), Syringic acid (+ 98%), and 4-Hydroxybenzoic acid (99%) were purchased from Alfa Aesar (The Netherlands), gallic acid (≥ 99.5%) was obtained from Loba Chemie Pvt. Ltd (India).

Tryptone was obtained from CONDA. Yeast extract was obtained from Bio Basic Canada INC. Deionized water was used in all experiments. All used solutions were prepared fresh before use. A flat sheet of commercial organic polyethersulfone (PES; 0.03 μm pore size as stated in the specification sheet) was obtained from Sterlitch (USA). PES polymer was obtained from BASF (Germany), Prime grade silicon wafers with a 2.5 nm native oxide layer were purchased from Wafer Net Inc (USA).

### Bacterial strains and media

A bacterial mixture of five strains was used to examine the modified membrane under similar real marine environment conditions. Three of them are biofilm-forming bacterial strains isolated from the Mediterranean Sea; *Acinetobacter* sp. EGY-WCA1 (KF576652.1), *Bacillus* sp. EGY-SC*R3 (KF217252.1) and *Pseudomonas stutzeri* EGY-WDB10 (KJ545605.1)^30^. In addition to *Escherichia coli* NCTC10418 (*E. coli*) and *Staphylococcus aureus* ATCC6538 *(S. aureus)* strains as models for Gram-negative and Gram-positive bacteria, respectively.

#### Artificial seawater (ASW) medium

The following composition (g/l): NaCl (27), MgSO_4_.7H_2_O (6.6), MgCl_2_.2H_2_O (6.5), CaCl_2_.2H_2_O (1.5), KNO_3_ (1.0), NaHCO_3_ (0.04). 20 ml of Tris-HCl buffer (1.0 M, pH 7.0) was added, 1.0 ml chelated ion solution, which composed of: FeCl_3_.4H_2_O, 240 mg/100 ml, EDTA, 14.6 g/100 ml and 1.0 ml. of Trace metal solution. Trace metal solution composition was (mg/100 ml): H_3_BO_3_ (60), MnCl_2_.4H_2_O (40), (NH_4_)Mo_7_O_24_.4H_2_O (37), CuCl_2_.2H_2_O (4), ZnCl_2_, (4), CoCl_2_.6H_2_O (1.5)^30^. All chemicals were obtained from Merck, Fluka and Riedel-de Haen.

#### Luria-Bertani (LB) liquid media

LB media was used as a culturing medium which contained 1% tryptone, 0.5% yeast extract, and 0.5% NaCl.

### Methods

#### Grafting modification of polyethersulfone membranes

A flat sheet of commercial polyethersulfone (PES) membranes was cut into circles with a diameter of one cm for evaluation of bacterial adhesion under various environmental conditions within a static fluid system. Moreover, these commercial membranes are used to evaluate the antibacterial compounds added to the 3-aminophenol modified PES membrane as a second modification layer to form a dual-layered membrane.

The modification parameters, including concentrations, enzyme units, and reaction durations, were carefully optimized based on prior research conducted by our team, which demonstrated their effectiveness in repelling bacterial adhesion. These selected conditions are not arbitrary; rather, they reflect the best-performing settings identified through extensive experimentation, as documented in several studies^6,7,10,31,32^. This optimization ensures the enhanced efficacy of the modified membranes in mitigating biofouling.

##### One-layered modification of PES membrane

The PES membrane circles were immersed in 40 ml of a 0.1 M sodium acetate buffer at pH 5, containing 15 mM of a specific aminophenol modifier (2-aminophenol; 2-AP, 3-aminophenol; 3-AP, or 4-aminophenol; 4-AP) along with laccase enzyme (0.5 U/ml). Air was introduced into the solution to facilitate mixing and to provide an oxygen source for the enzymatic reaction. This reaction was conducted at ambient temperature (23 ± 2 °C). Following the designated modification period (15–30 min), the PES membranes were washed by immersion in boiled distilled water three times, followed by three immersions in cold distilled water. Subsequently, the samples were dried for 72 h in glass-covered dishes in desiccators containing silica gel^9,31^.

##### Dual-layered modification of PES membrane

The one-layer modified PES membrane (3-AP/PES) was used to add the second layer of the antimicrobial agents by using the same previously described method (the enzyme-catalyzed modification method). 4-Hydroxybenzoic acid (B), gallic acid (G), syringic acid (S), and vanillic acid (V) were employed to impart antibacterial properties to the 3-AP/PES membranes, which were tested for 30 min. The antibacterial layers were prepared using separate solutions of 15 mM of each compound in a 0.1 M sodium acetate buffer at pH 5, containing laccase enzyme at a concentration of 0.5 U/ml. For G, the modification duration was set at 2 h, whereas for B, S, and V, a 24 h modification period was employed^12,33^. It was proven that prolonged grafting times with G resulted in increased intra-layer reactivity, enhancing cross-linking and network formation, which ultimately led to more compact layers. This compaction, in turn, heightened protein adsorption, thereby facilitating the onset of biofouling^6^.

**Fig. 4:**
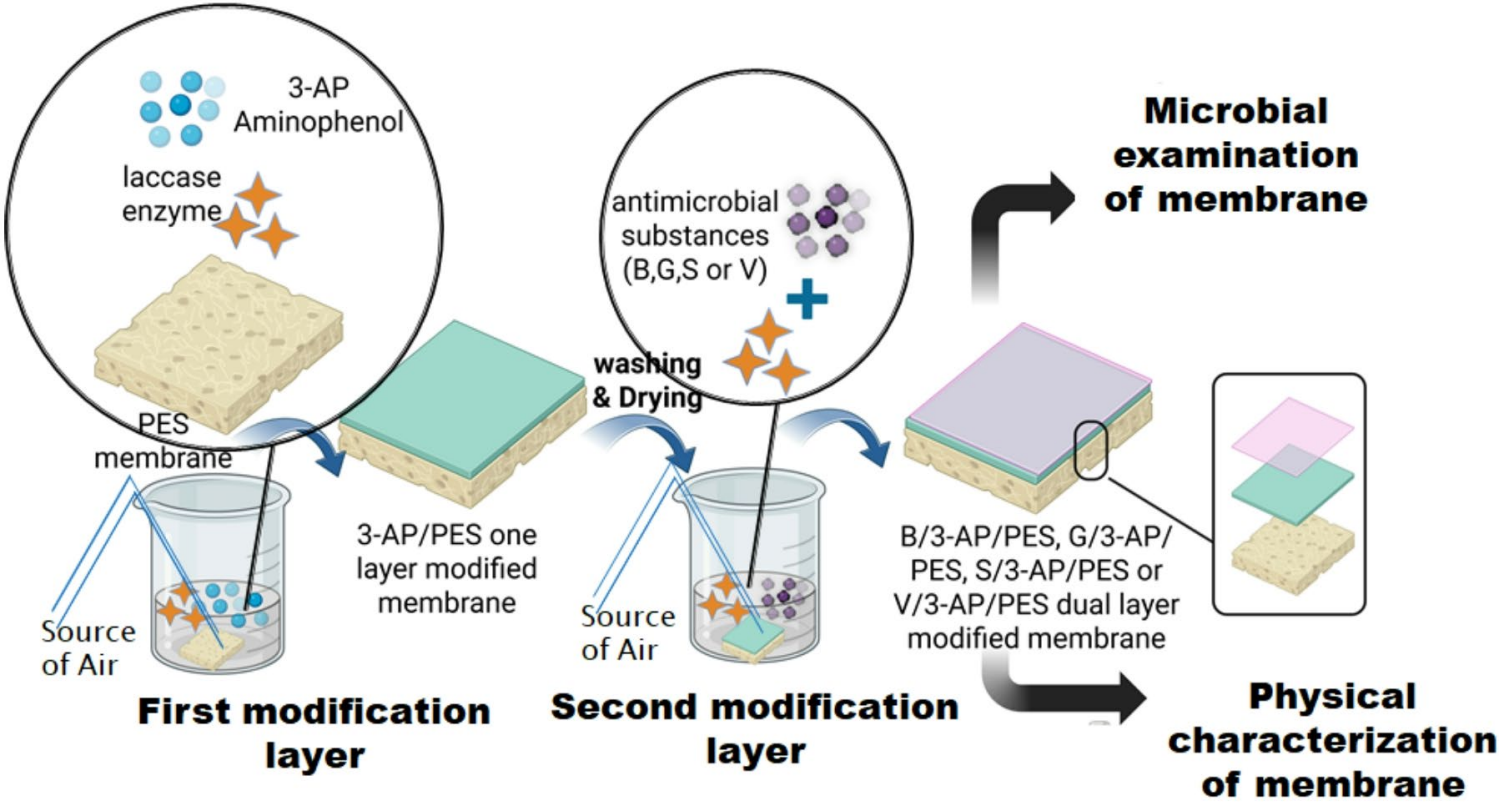
Schematic diagram of the one-layer and dual-layer enzyme-catalyzed modification process using polyethersulfone(PES) membrane and 3-aminophenol (3-AP), 4-hydroxybenzoic acid (B), gallic acid (G), syringic acid (S), and vanillic acid (V) modifiers (Created in www.BioRender.com).

The antibacterial agents (B, G, S, and V) were dissolved through a combination of stirring and heating. The enzymatic reaction occurred at ambient temperature (23 ± 2 °C), with air being bubbled through the solution to ensure mixing and provide a source of oxygen. Upon completion of the incubation period, the dual-effect modified PES membranes were washed three times by immersion in cold distilled water. Subsequently, the samples were dried for 72 h in glass-covered dishes placed within desiccators containing silica gel^6^. Table 1 shows the codes used for the blank and the modified one and the dual-layered membranes. Figure 4 shows the steps of one-layer and dual-layer enzyme-catalyzed modification of PES membrane.

**Table 1:** Membrane codes and their layers.

Membrane Code	Modification Layers
PES (Blank)	unmodified polyethersulfone
3-AP/PES	3-aminophenol/polyethersulfone
B/3-AP/PES	4-hydroxybenzoic acid/3-aminophenol/polyethersulfone
G/3-AP/PES	gallic acid/3-aminophenol/polyethersulfone
S/3-AP/PES	syringic acid/3-aminophenol/polyethersulfone
V/3-AP/PES	vanillic acid/3-aminophenol/polyethersulfone

####  Biofilm development and assessment of the modified membranes across varying operational conditions

A bacterial mixture was employed in these experiments, comprising *E. coli*,* S. aureus*,* Acinetobacter* sp., *Bacillus* sp., *and P. stutzeri*. Various media were tested to identify the most suitable for bacterial growth while simulating seawater content. Amer & Fattah’s ASW (2014) was selected to determine the most effective formulation. Phosphate-buffered saline (0.1 M PBS, pH 7) was utilized for serial dilutions in the total plate count assay. Overnight bacterial cultures were generated by inoculating 5 ml of LB broth with a loopful of stock cultures from each strain separately, followed by incubation in a shaker incubator at 150 rpm and 30 °C for 24 h^30^. The bacterial culture was then centrifuged (Hermle, Labnet Z206 A) at 6000 rpm for 15 min. The supernatant was discarded, and the pellet was re-suspended in one ml of fresh LB broth to make stock bacterial culture of optical density (OD_600_) 10.

This segment of the study was conducted within a static fluid system to facilitate the exposure of the modified membranes to a higher bacterial load. The tubes were incubated for 48 h to enhance the likelihood of bacterial adhesion to the PES membranes. Modified PES membranes treated with 2-AP and 3-AP for durations of 30 min, but 4-AP for 15 min, which were identified as the most effective for mitigating biofouling. A mixture culture comprising of *Acinetobacter* sp., *Bacillus* sp., *and P. stutzeri* —salt-tolerant bacteria isolated from Mediterranean environments—along with *E. coli and S. aureus*, was employed to assess the efficacy of the modified membranes in resisting biofouling. Quantitative analysis of biofilm formation was performed using the microtiter plate method^34^, with measurements taken using a micro-ELISA auto reader (BioTek) at a wavelength of 630 nm. Artificial ASW was utilized to replicate marine environmental conditions, with its composition closely mirroring the chemical constituents of seawater used in the desalination processes^30^.

Circles with a diameter of one cm of both modified and unmodified commercial PES membranes were utilized in the subsequent experiments. ASW mixture of *Acinetobacter* sp., *Bacillus* sp., *P. stutzeri*,* E. coli*,* and S. aureus* was inoculated in LB media to establish an overnight culture. It was subcultured in ASW medium to achieve an optical density of approximately 0.07^35^. The ASW media were used with varying salinity levels (35, 42, and 57 ppt NaCl) at pH values of 7.5, 8, or 8.5. The pH of the solutions was regulated using 0.1 N HCl and 0.1 N NaOH. Each solution was dispensed in 4 ml aliquots into the wells of a sterile 12-well flat-bottom tissue culture plate. Two triplicate groups of both unmodified and modified membranes were placed separately in the wells of two tissue culture plates. These plates were incubated for 48 h at temperatures of 15 °C, 25 °C, or 35 °C in a static refrigerated incubator (Innova, model 4230, USA) to facilitate biofilm formation on the membranes. In each experiment, one variable was changed while the other factors remained constant.

##### Biofilm formation and membrane harvesting

Following the incubation period, the contents of each well, along with the PES membranes, were prepared to assess the efficacy of the method in detecting biofouling on membranes. The contents from each well were carefully transferred to sterile Falcon tubes to quantify biofilm formation and determine the optical density of bacterial growth via spectroscopy at OD_600_^36^. The PES membranes were subsequently moved to a clean and sterile 12-well microtiter plate (MTP) and subjected to two washes with 6 ml of sterile distilled water, agitated at 120 rpm for 15 min, to eliminate loosely adhered bacteria (planktonic bacteria). Triplicate membranes were placed in 3 ml of phosphate-buffered saline (PBS) at pH 7.3 and individually subjected to ultrasonication for 3 min using a Q500 sonicator (Vibra-cell ^TM^ Ultrasonic Liquid Processor VCX 130, Sonics & Materials, Inc., Newton, CT, USA) at 25% amplitude with 2-second pulsating intervals to facilitate biofilm disassembly into the PBS suspension^36^.

##### Evaluating the biofilm formation under different operational conditions

Various methods for biofilm detection were evaluated under static conditions in our study to establish a multi-faceted approach for cross-confirmation. These methods include bacterial load assessment (through optical density, hemocytometer by microscopy, and scanning electron microscope images), in addition to viable bacterial counting via TPC. All experiments were conducted with three replicates (*n* = 3), and the results are presented as the mean ± standard deviation for all quantitative data.

##### Bacterial load investigations methods

Following the incubation period of the bacterial mixture under the specified operational conditions, which leads to biofilm formation on the membranes due to the accumulation of adhered bacterial cells.

##### Optical density (OD) method

The bacterial concentration in the solution was monitored through optical density measurements using a visible spectrophotometer (Novaspec II). The growth of bacterial cultures is assessed by measuring their optical density at OD_600_^37^.

##### Viable bacterial count by the total plate count (TPC) method

In the Total Plate Count method, serial dilutions in phosphate-buffered saline (PBS) were performed, and 100 µl from the most appropriate dilutions for culturing were inoculated onto LB agar plates. The enumeration of colonies was conducted on the LB agar plates to ascertain the bacterial concentration expressed as Colony Forming Units (CFU) following a 48 h incubation period at 35 °C in a static incubator (Innova, model 4230, USA)^38^.

##### 3 M™ Petrifilm aerobic count plates method

3 M™ Petrifilm AC plates identified organisms in a shorter timeframe than alternative methods. The 3 M™ Petrifilm aerobic count plate (3 M, St. Paul, MN) is a pre-prepared medium designed specifically to quantify total aerobic bacterial populations^39^.

##### Hemocytometer measurement assay method

One aliquot from each diluted sample was subjected to a hemocytometer count to quantify bacterial cell counts in comparison to red blood cell counterparts. The results indicate the number of bacterial cells present in each concentration of the tested sample. Representative wells were photographed using an inverted microscope equipped with a camera^40^. Four images from random locations were captured, and the cell count was analyzed using ImageJ software.

##### Scanning electron microscope (SEM) imaging

Biofilm formation and attached bacteria on the membrane surface were estimated by using scanning electron microscopy (SEM, JSM-200 IT, Japan). The membrane samples were gently washed and dried till they reached to critical-point drying. Then the membranes were coated with gold using a sputter-coating system (Ion sputtering device, JEOL, JFC-1100E, Japan) and were examined at various magnification powers (2000X, 5000X and 10000X), operating at fixed voltages of 20 kV^31^. Pore size was calculated using SEM images by imageJ software.

#### Study the impact of four selected antibacterial compounds on the inhibition of bacterial growth

The 3-aminophenol (3-AP) modified membrane exhibited an effective capacity to inhibit bacterial attachment, which could be attributed to its brush-like structure formed in the modification layer, as previously described. Four compounds were employed to achieve a dual antibacterial and anti-attachment effect by creating a second modification layer on the 3-AP/PES membrane. The used compounds were 4-hydroxybenzoic acid (B), gallic acid (G), syringic acid (S), or vanillic acid (V). They were tested for their antibacterial efficacy at concentrations of 1, 5, 10, 15, and 28.8 mM as separate monomer compounds.

Different concentrations of vanillic acid (V) (1, 5, 10, and 15 mM) were prepared in 25 ml of sterile LB media to determine the minimum inhibitory concentration (MIC). Subsequently, 175 µl of the enriched bacterial stock cultures were inoculated to achieve an optical density (OD_600_) of approximately 0.07 in the final subculture following the method of Liu *et al*. (2018)^35^. The cultures were then incubated in a shaker with 150 rpm at 30 °C for 24 h. The optical density of the subcultures was recorded, and serial dilutions in PBS were prepared. From the appropriate dilution, the optical density and total bacterial count were tested. The same effective concentration of V was applied to the other three antibacterial agents, and bacterial growth inhibition was assessed in the same manner. It is noteworthy that solutions of G and V required heating and constant stirring for 4 h to ensure complete dissolution^41^.

#### Assessment of antibacterial properties of the dual-layer modified membranes

A bacterial mixture comprising five selected strains was inoculated in ASW media, containing 35% ppt salinity and adjusted to pH 7.5, at 25 °C for 48 h, utilizing the 3-Aminophenol (3-AP) modified membranes subjected to a 30 min treatment. Microbial growth was monitored by optical density (OD), total plate count (TPC), and hemocytometer count. In the preceding section, microbial examination was performed using the conventional plating; however, in this part, TPC was evaluated using the 3 M™ Petrifilm™ plate method, followed by incubation at 36 °C for 48 h to develop the color on the films^42^.

#### Physical characterization of the dual-effect modified membranes

##### **The grafting yield**

Defined as the amount of added modifier per unit area. The membranes were kept for 72 h in covered dishes in a desiccator to remove any moisture^12^.

**Scanning Electron Microscoy (SEM) imaging. **The cross-section and surface of the membranes were examined using scanning electron microscopy (SEM). For the cross-section examination, the dual-layer modified membranes samples were cut with a sharp shaving blade and dried to critical-point drying. Following this, the membranes were coated with gold using a sputter-coating system (Ion sputtering device, JEOL, JFC-1100E, Japan) and examined at various magnification powers, with a resolution of 1280 × 960 pixels.

**The static water contact angle.** The hydrophilicity of the membrane surface was assessed through contact angle measurements utilizing a contact angle goniometer (ramé-hart instrument co., model 190, Tiba Scientific, US) in conjunction with DROP image CA v2.5 software. The static water contact angles of both unmodified and modified PES membranes were determined by depositing a drop of demineralized water on five distinct locations of each membrane. All parameters were calculated as the average of five readings taken from each membrane sample after a duration of 5 s.

**Fourier Transform Infrared-Attenuated total reflection (FTIR-ATR)** spectroscopy bands were recorded at a resolution of 1 cm⁻¹ using a BRUKER ALPHA II transmission spectrophotometer (Germany). The spectral range was captured as a function of wavelength, spanning from 400 to 4000 cm⁻¹.

##### Atomic force microscopy (AFM) imaging. 

The blank and modified PES surfaces were represented on strips, and images were acquired using a WITec alpha300 R atomic force microscope (Germany). AFM was utilized to ascertain roughness parameters by extracting the root-mean-square (RMS) roughness, deemed the most reliable indicator of surface corrugation. The RMS profiles, indicative of roughness, were calculated for both samples before and after the second modification, to calculate RMS roughness using the free data analysis software Gwyddion^33^. Surface Area Difference (SAD) reflects the total three-dimensional surface area, which typically increases with roughness. SAD calculated as SAD = [(Am – Asc)/Asc] × 100, where Am is the measured surface area and Asc is the imposed scanned area^43^.

#### **Statistical analysis**

The effects of temperature, pH, and salinity on membrane biofouling were assessed using analysis of variance (ANOVA) under a completely randomized design (CRD) with three response variables: optical density (OD_600_), total plate count (TPC), and hemocytometer bacterial cell counts. Data analysis was executed using Statistix 10.0 (USA) software. The significance level was established at *p* < 0.05. Post hoc comparisons for all analyses were conducted using Tukey’s HSD multiple comparison tests at α = 0.05 to explore the relationships among all group combinations.

## Results and discussion

*E. coli and S. aureus* were chosen as representative models for Gram-negative and Gram-positive bacteria, respectively, in conjunction with a mixture of salt-tolerant bacteria. *S. aureus* is one of the most prevalent bacteria in implant-associated infection, and its microbial surface components contain adhesive matrix substance^27^. The isolates *Acinetobacter* sp., *Bacillus* sp. and *P. stutzeri*, were considered biofilm-forming bacteria positive if they had an OD_570_ of 0.12. If the optical density (OD_570_) exceeded 0.24, they were classified as strongly adherent^30^. The salt-tolerant bacterial isolates were screened for their ability to form biofilm by MTP method^44^. The optical density values obtained from the biofilm were regarded as indicators of bacterial attachment to the surface and subsequent biofilm formation^34^. OD_630_ turbidity. These organisms were inoculated in artificial seawater (ASW) media. A variety of evaluation procedures were employed to assess the efficacy of each modification model. ASW emerged as the most reliable and suitable medium for our experimental aims, as it elevated the optical density at OD_600_ from 0.01 to 1.4 after 48 h of incubation. Previous findings indicate that static conditions yield a higher bacterial load on membranes compared to dynamic conditions; therefore, static conditions were utilized in all subsequent experiments to investigate the efficiency of modified membranes in repelling bacterial adhesion^32^.

### Assessment of biofilm formation and bacterial repellence of one-layer modified membranes under varing temperature conditions

A mixture of *Acinetobacter* sp., *Bacillus* sp., *P. stutzeri*, *S. aureus*, and *E. coli* was cultured in ASW media at pH 7.6 and total dissolved solids (TDS) of 35 ppt for a 48 h incubation at 15 °C, 25 °C, and 30 °C. The initial overnight culture for the 15 °C, 25 °C and 35 °C experiments exhibited an optical density (OD_600_) of 0.141, 0.116 and 0.078, respectively. Optical density measurements, total bacterial counts, and hemocytometer counts were conducted to evaluate biofilm formation. Total viable count (TPC) assessed live cells, whereas both OD_600_ and hemocytometer counts included both viable and non-viable cells. Achieving reproducible biofilm formation on PES membranes proved to be exceedingly challenging^38^. Numerous assays exist for quantifying biofilm formation on surfaces, yet only a few methodologies can effectively assess the antibiofilm efficacy of agents against biofilm development. It has been established that the Total Plate Count (TPC) method alone is inadequate for detecting bacterial biofilm due to its substantial variability. Thus, the primary objective of this segment of the research is to provide an overview of techniques for assessing biofilm formation. Petrifilm AC plates provided reproducible results in comparison to traditional techniques and are well-suited to meet the rapid industrial demands for example, milk quality control^39^.

**Fig. 5:**
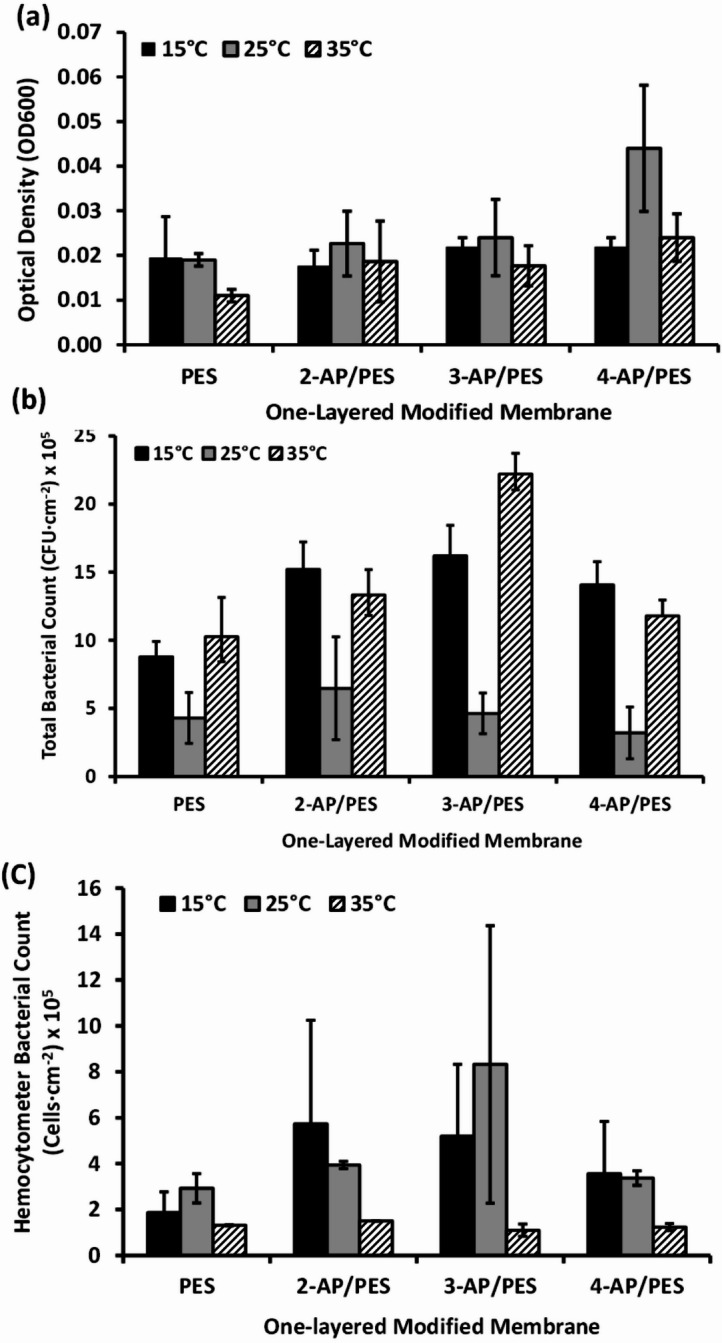
Microbial examination (**a**) Optical Density (OD_600_), (**b**) total bacterial count, and (**c**) hemocytometer counts, were conducted to investigate the effect of varying temperatures (15 °C, 25 °C, and 35 °C) on the bacterial repellence of an unmodified polyethersulfone (PES) membrane and three modified ones (2-AP/PES, 3-AP/PES, and 4-AP/PES).

Our findings suggest that 25 °C and 35 °C are the most conducive temperatures for nearly all aminophenol-modified membranes in terms of minimizing bacterial attachment and biofilm development, as illustrated in Fig. 5. That anti-attachment property may be produced as a result of mass transfer enhancing and increasing the permeate flux by elevating the temperature, which mitigates particle accumulation and biofilm deposition. Conversely, Fig. 5 indicates that all aminophenol-modified membranes were ineffective in reducing bacterial adhesion at 15 °C, highlighting their limited efficacy in colder climates. Notably, the 3-AP modified membrane exhibited commendable biofouling resistance at 25 °C and 35 °C, significantly lowering both viable and non-viable cell counts. Additionally, the 4-AP modified membrane demonstrated an inhibition of the viable bacterial cell count in TPC assessments across all tested temperature conditions. This fully agrees with previous work suggests that 4-AP does not form a poly(4-AP) polymeric layer. Nevertheless, it forms monomers or dimers of quinone and the free hydroxyl groups that maintain its antibacterial phenolic characteristics. The greater bactericidal activity of 4-AP is assumed to be a result of its ability to diffuse across the bacterial cell walls and the cytoplasmic membrane of bacteria^32,45^.

As referenced in prior research, a decline in temperature to 15 °C correlated with an increased fouling rate of flat-sheet PES membranes, exacerbated by the accumulation of microbial byproducts and inorganic foulants, along with elevated levels of extracellular polymeric substances (EPS), polysaccharides, and proteins in the supernatants^46^. According to Pichardo-Romero, Garcia-Arce *et al.* (2020) study, at 15 °C the larger size of colloids (organic foulant) formed a cake layer deposition and higher flux decline, as well which was not formed at 25–35 °C^13^. In spite of that, other authors concluded that nanofiltration polyamide membranes at higher temperatures showed less rejection of foulants, and flux decline due to increasing polymer chain mobility which resulted in changing membrane properties. Thus, most water treatment technologies provide optimal performance at 25 ± 2 °C^47^.

### Assessment of biofilm formation and bacterial repellence of one-layer modified membranes under varied pH conditions

The pH can significantly influence the physicochemical properties of membranes, thereby altering their fouling behavior. Five bacterial strains were incubated in ASW media at pH levels of 7.5, 8.0, and 8.5, with a total dissolved solids (TDS) concentration of 35 ppt, over a 48 h period at 25 °C. The initial overnight culture for pH 7.5 optical density (OD_600_) was 0.116, where for pH 8 it was 0.117, for pH 8.5 it was 0.081.

**Fig. 6:**
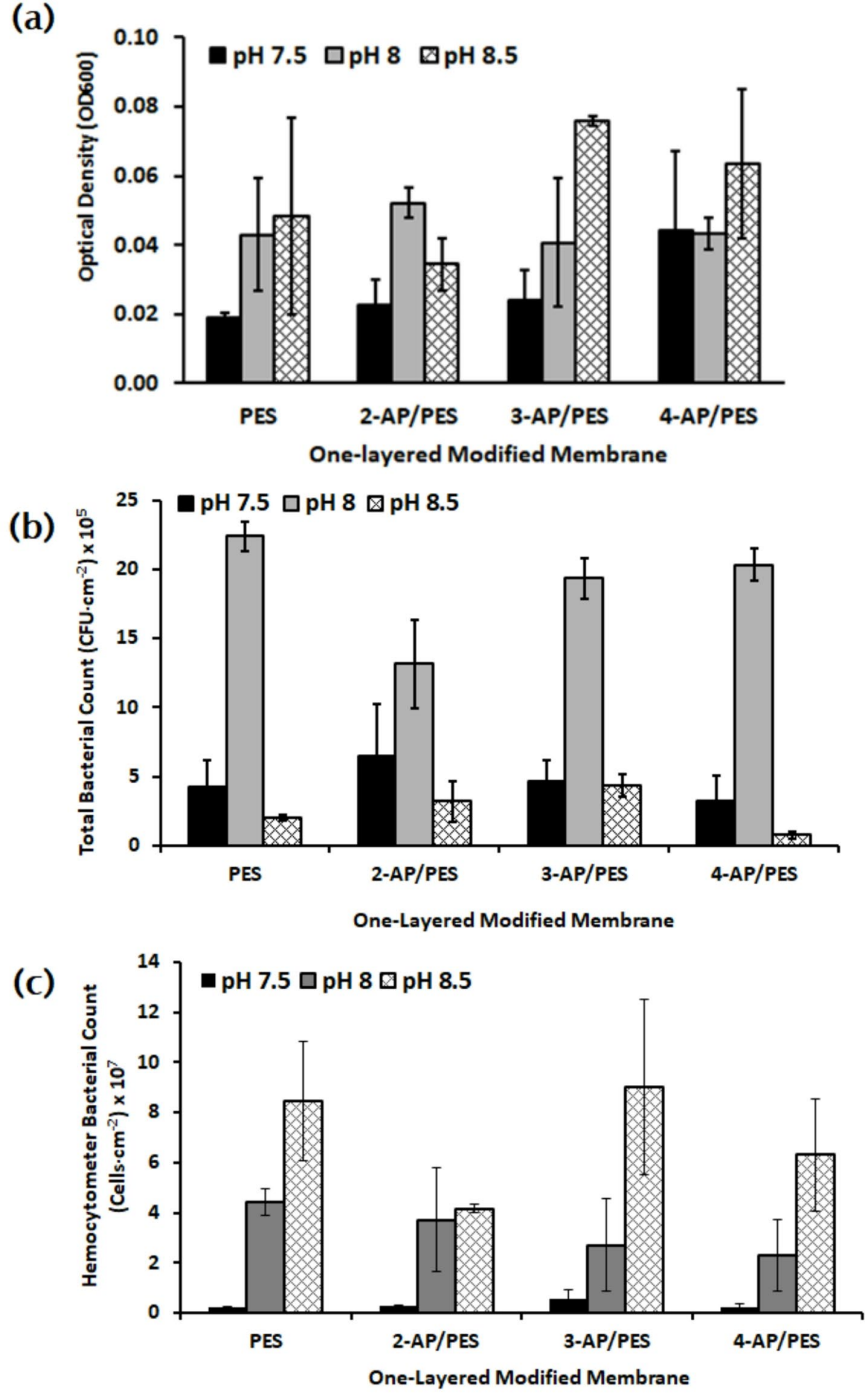
Microbial examination (**a**) Optical Density (OD_600_), (**b**) total bacterial count, and (**c**) hemocytometer counts, were conducted to investigate the effect of varying pH levels 7.5, 8.0, and 8.5on the bacterial repellence of an unmodified polyethersulfone (PES) membrane and three modified ones (2-AP/PES, 3-AP/PES, and 4-AP/PES).

The pH of seawater typically ranges from 7.5 to 8.5, making it slightly alkaline. Figure 6 illustrates the effect of 7.5, 8.0, and 8.5 on the bacterial repellence. The acidic and basic components significantly impacted the membrane fouling behavior over a short range. Notably, pH 7.5 demonstrated the most pronounced bacterial repellence compared to pH 8.0 and 8.5. In fact it was reported that the bacterial growth and activity along with efficiency of enzymes in biofilm can be influenced by pH values due to the dissociation of functional groups^13^. All biofilms in Schultze *et al.* (2020) study had lower total bacterial count at acidic pH than at higher pH values, despite the metabolic activity was highest at pH 8^48^. However, alkaline solutions increase the negative charge by increasing the solution pH and solubility of the organic foulant, in addition alkaline solutions clean organic-fouled membranes by hydrolysis and solubilization^49^. At pH 8.5, there were a lot of attached dead cells but low living bacterial load because dead cells may shrink and their size is reduced, which helps in internal pore-blocking and more strongly attachment of many dead cells as referred in Park* et al*. (2023)^50^.

Further, the 2-AP/PES membrane emerged as the most effective in repelling bacteria across nearly all pH values, as shown in Fig. 6. These results offer crucial insights for strategies aimed at mitigating membrane fouling. Gao *et al.* (2014) declared that fouling potential for organic foulants of microfiltration and ultrafiltration membranes decreased with increasing pH values in the order of: pH 5 > pH 7 > pH 8 > pH 9^51^. Also, the results of Donose *et al.* (2013) showed that the water permeability tests conveyed a noticeable reduction at pH 7 and pH 4 medium as long as increasing the ageing of the RO membranes^43^.

### Assessment of biofilm formation and bacterial repellence of one-layer modified membranes under different salinity levels

**Fig. 7:**
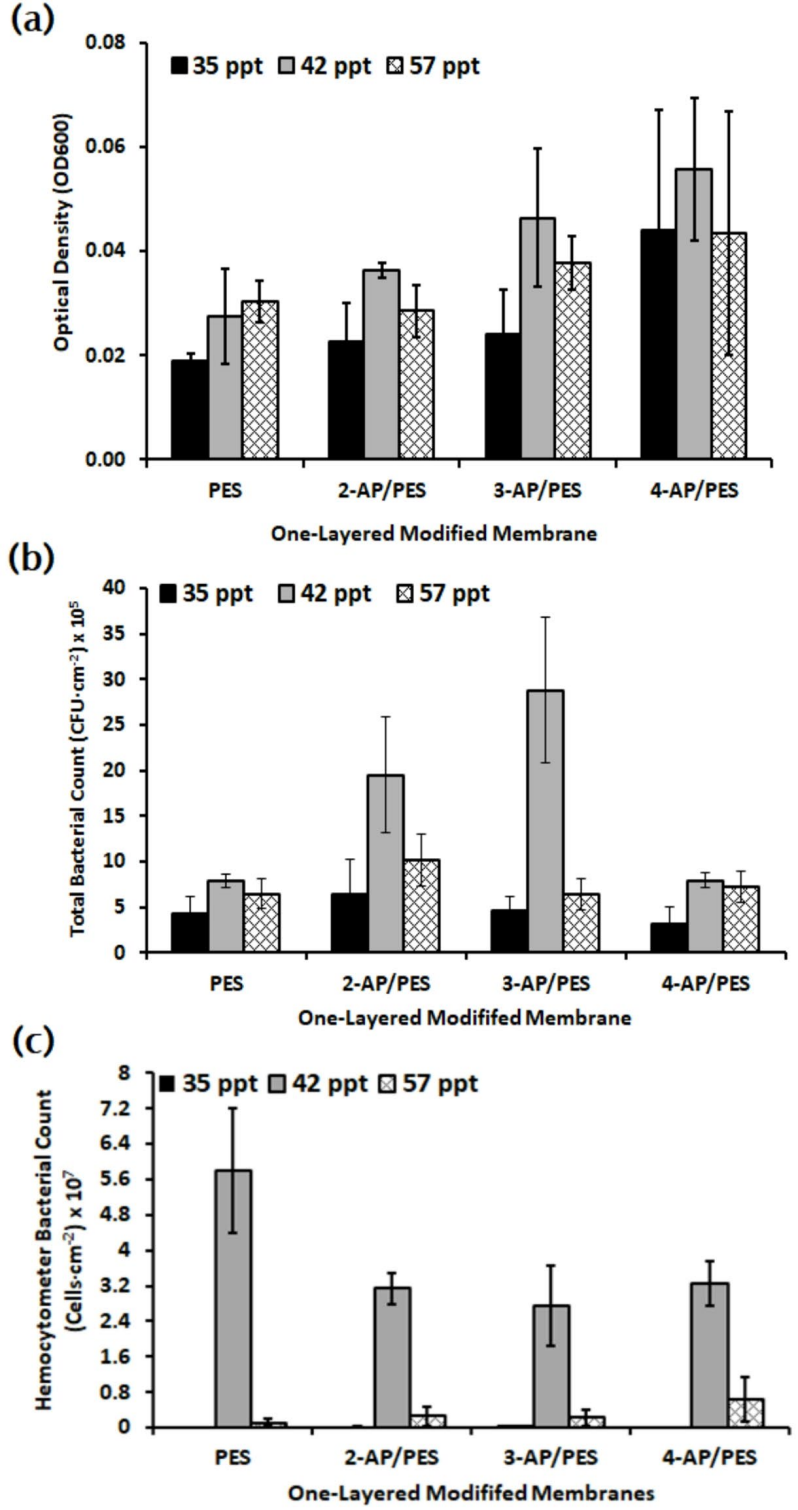
Microbial examination (**a**) Optical Density (OD_600_), (**b**) total bacterial count, and (**c**) hemocytometer counts, were conducted to investigate the effect of varying salinity levels 35, 42, and 57 ppt on the bacterial repellence of an unmodified polyethersulfone (PES) membrane and three modified ones (2-AP/PES, 3-AP/PES, and 4-AP/PES).

The initial overnight culture of the bacterial mixture for the 35 ppt experiment exhibited an absorbance of 0.1. In contrast, the absorbance values for the 42 ppt and 57 ppt experiments were recorded at 0.137 and 0.110, respectively. The treatments 35 ppt and 57 ppt salinity enhanced the bacterial repellence in membranes. Variations in the ionic strength of the solution significantly influence the extension of polymer chains^2^. Furthermore, elevated ionic strengths combined with acidic pH levels fostered bacterial adhesion by altering the membrane surface characteristics, particularly in terms of pore size and morphology^13^. Conversely, when bacterial cells are exposed to high external osmotic pressure, water is drawn out of the cells, leading to a decrease in cytoplasmic volume and, subsequently, a reduction in cell size. Notably, *E. coli* is not the sole organism capable of traversing membrane pores smaller than its own dimensions; this selective transfer can occur, as evidenced by the salinity of ASW at 42 ppt, as shown in Fig. 7.

However, the addition of both Na and Ca ions enhances fouling by the formation of a dense cake layer on the membrane, as happened in the 42 ppt experiment^2^. The high salinity increased the amount of attached extracellular polymeric substances (EPS) within the suspended sludge and resulted in severe membrane fouling^11^. When Na ion concentration increased at 57 ppt, the attached bacterial cell decreased as shown in Fig. 7, which was compatible with Charfi *et al.* (2017) findings. They pointed out the addition of Na or Ca ions to a solution mitigates membrane fouling^2^. While salinity inhibited bacterial proliferation and EPS secretion, the relative abundance of polysaccharides within the fouling layer increased^20^. The response of quorum sensing (QS) to salinity stress plays an important role in regulating biofouling behavior. Results of Song *et al.* (2024) showed that high salinity stress (over 20 g/l of NaCl) greatly stimulated QS activity, acyl-homoserine lactones (AHLs) were increased by over 200% in the cake layer and showed strong correlations with changes of fouling tendency and EPS characteristics^52^. Moreover, membranes modified with 4-AP/PES demonstrated the highest efficacy in repelling living bacterial cells across all salinity levels during the TPC experiment due to its bactericidal effect of dimers, whereas 2-AP/PES and 3-AP/PES exhibited superior bacterial repellence for both living and dead cells as it was reported in almost all salinity levels.

Based on the data derived of temperature, pH, and salinity the modified PES membranes incorporating 2-AP/PES, 3-AP/PES, and 4-AP/PES were found to be useful in the Mediterranean region. The 3-AP/PES membrane treated for 30 min gave results for mitigating biofouling across a wide range of operational conditions. Furthermore, the brush-like structural polymerization of 3-AP/PES plays a crucial role in protecting the membrane’s integrity and enhancing its biofouling resistance as mentioned by our previous findings^8,32^. Consequently, it was employed in the evaluation of dual-layer antibacterial PES membranes.

### The minimum inhibitory concentration (MIC) of the four antibacterial agents

According to the structural similarity of vanillic acid (V) (4-hydroxy-3-methoxybenzaldehyde) to other antimicrobial substitutes, the initial investigation focused on the effects of varying concentrations of V at 0, 1, 5, and 10 mM on the bacterial growth of *S. aureus*, *E. coli*, and a salt-tolerant bacterial mixture, to determine the minimum inhibitory concentration (MIC). At a concentration of 10 mM, no visible bacterial growth was observed in any of the cultures after a 24 h incubation at 37 °C. Furthermore, the polymerization reaction involving V necessitates a high concentration of the monomer to facilitate a noticeable color change; thus, V was selected to identify the most effective concentration of monomers for mitigating bacterial growth^12^.

**Fig. 8:**
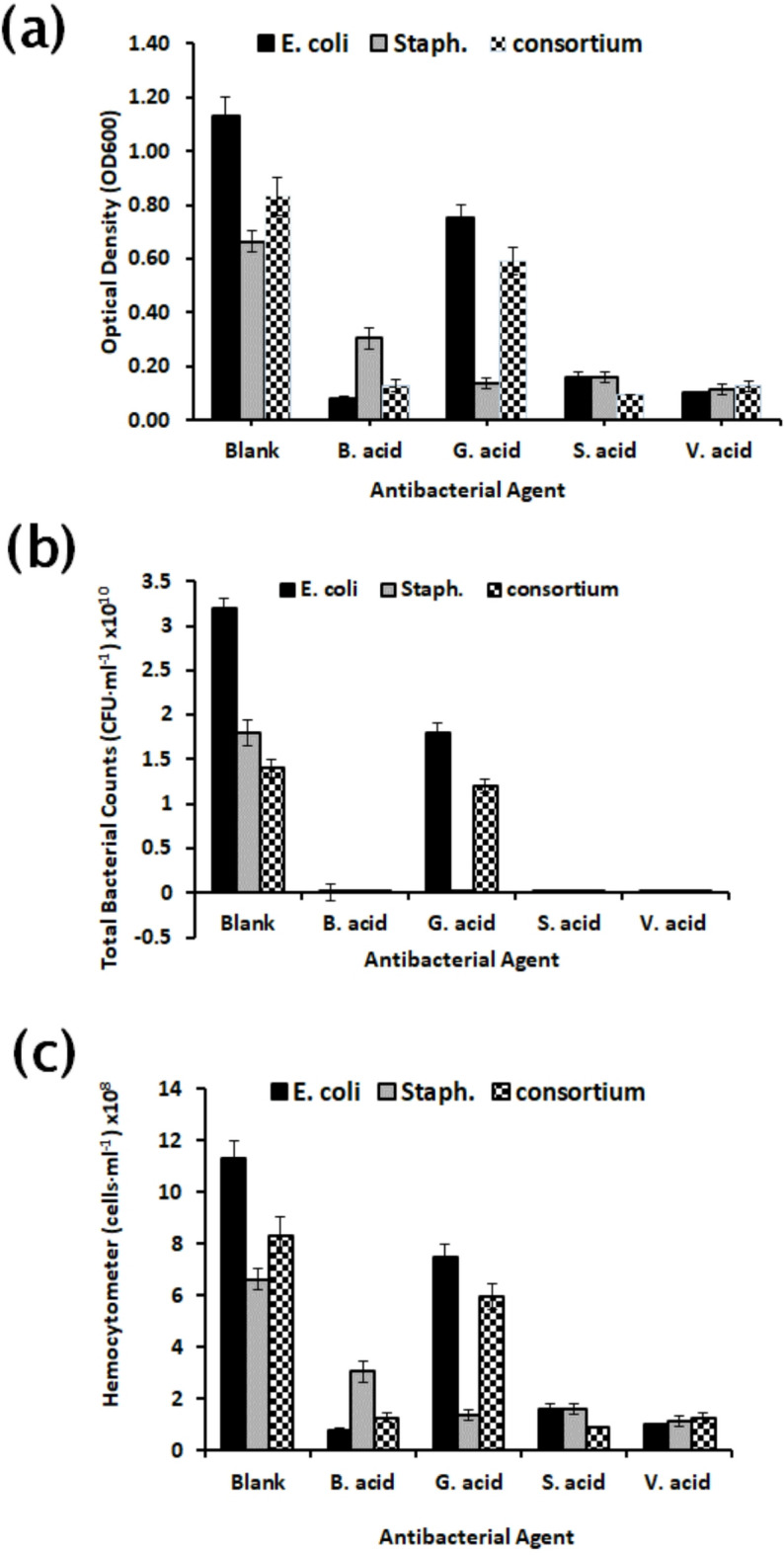
Minimum Inhibitory Concentration (MIC) evaluation of antimicrobial agents: (B) 4-hydroxybenzoic acid, (G) gallic acid, (S) syringic acid, and (V) vanillic acid, compared with a control (Blank). Antibacterial activity was assessed against a bacterial mixture using (**a**) optical density (OD_600_), (**b**) total plate count (TPC), and (**c**) hemocytometer count.

The four antibacterial monomers (4-hydroxybenzoic acid (B), gallic acid (G), syringic acid (S), or vanillic acid (V)) substitutes with 15 mM concentrationdemonstrated a promising antibacterial effect against the growth of the bacterial mixture, particularly compared to the control as shown in Fig. 8. Notably, B, S, and V exhibited the most pronounced bactericidal effects, especially against the salt-tolerant bacteria. In agreement with another study, B, G, S, and V could inhibit bacterial growth and some metabolic activities in *E. coli* by collapsing ion gradients and increasing internal anion concentrations and their MICs were 40, 15, 17.5 and 15 mg/ml, respectively^25^. In Liu* et al*. (2017) study, the MIC of G against *S. aureus* in suspension and in biofilms was 2 and 4 mg/ml, respectively^24^, so in biofilm inhibition determination experiment, the concentration of phenolic compounds is preferred to be increased than MIC which influences bacterial growth only. Shao *et al.* (2015) indicated that G with 8 mg/ml could inhibit the bacterial growth and biofilm formation of Gram-negative (*E. coli)* more than Gram-positive (*Streptococcus mutans)* which is more resistant to G^53^.

### Assessment of biofilm formation and bacterial repellence of dual-layer modified PES membranes

The PES membrane, initially modified with 3-AP, was subjected to a second modification step in order to produce dual-layered membranes. The modification conditions were examined, and it was found that 15 mM modifier concentration with 30 min reaction time was sufficient to achieve a predominantly bonded layer. It was reported earlier by our group that at higher monomer concentrations, a significant fraction of the grafted material is incorporated as non-covalent homopolymers through physisorption, which do not provide stable covalent bonding to the membrane^33^.

The reactivity of the selected phenolic modifiers varied considerably. Gallic acid (G), which possesses two additional hydroxyl groups compared to 4-hydroxybenzoic acid (B), has demonstrated higher reactivity and faster grafting kinetics^54^. At low G concentrations, the color change was less pronounced with increasing reaction time, suggesting that the available G molecules were already fully consumed during the initial stages of the reaction. By contrast, vanillic acid (V) reacted at a much slower rate, and color changes were only evident after 48 h at high monomer concentrations. Syringic acid (S) did not produce any observable membrane color change of the membrane surface, even when high monomer and enzyme concentrations or extended modification times were applied^12^. To ensure that the modification reaction had occurred, both grafting yield and physical characterization techniques were employed, indicating successful surface modification.

**Fig. 9:**
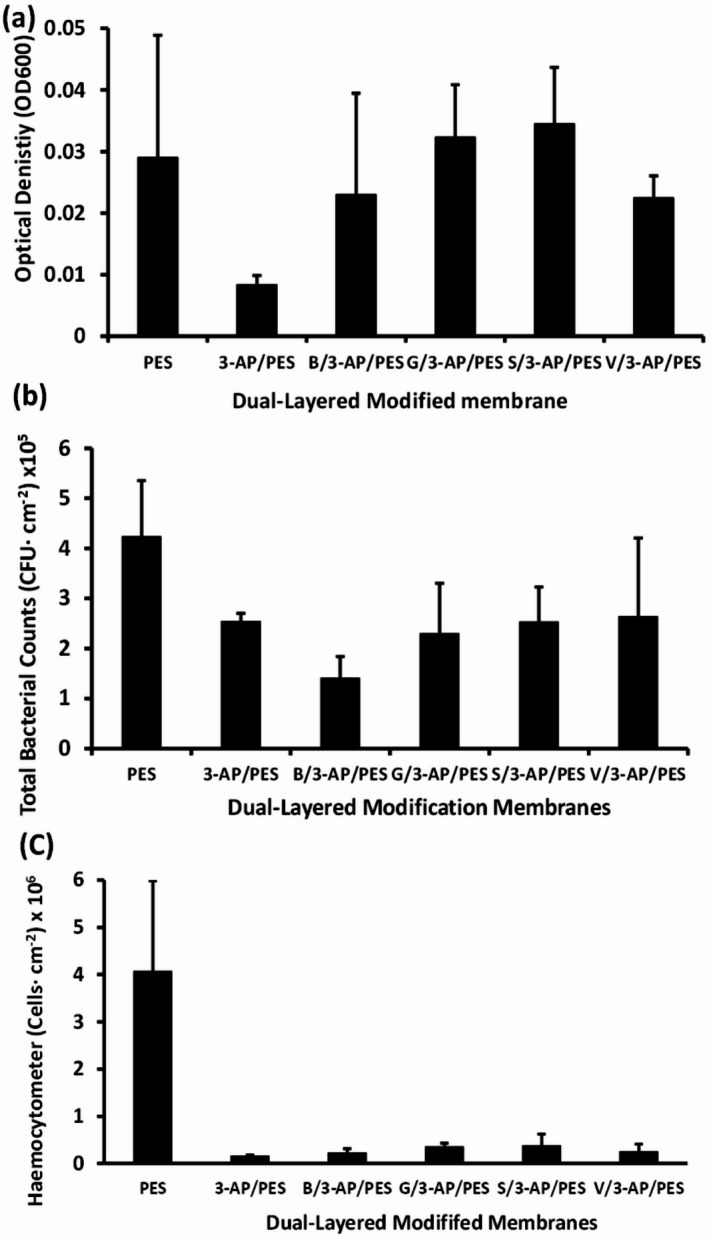
Antimicrobial effect of dual-layered PES membranes (B/3-AP/PES, G/3-AP/PES, S/3-AP/PES, V/3-AP/PES) compared with PES and 3-AP/PES. Tests were conducted against a bacterial mixture in ASW medium at 25 °C for 48 h, with efficacy assessed by (**a**) OD_600_, (**b**) TPC, and (**c**) hemocytometer count.

The dual-layered membranes; B/3-AP/PES, G/3-AP/PES, S/3-AP/PES and V/3-AP/PES were evaluated against the bacterial mixture. The 3-AP/PES membrane demonstrated the higher inhibition of bacterial growth, as reflected by a 72% reduction in bacterial density (OD_600_) of detached biofilm and a 96% reduction in cell counts determined via hemocytometry, encompassing both viable and non-viable populations as shown in Fig. 9. B/3-AP/PES membrane achieved the highest suppression of viable cell attachment, with a 77% reduction confirmed through total plate count (TPC) analysis. This was attributed to the bactericidal effect of B, which killed cells, thus increasing the overall count of non-viable bacteria. The most effective antibacterial performance of B/3-AP/PES may be explained by the presence of quinone and hydroxyl group dimers in B, which maintain strong phenolic antimicrobial characteristics as evidenced before by Nady *et al.* (2012)^33^.

In contrast, G/3-AP/PES displayed relatively weak biofilm inhibition, with higher bacterial loads observed (Fig. 9). This is consistent with earlier reports showing that G exhibits significant antibiofilm properties only at higher concentrations. For example, Shao *et al.* (2015) demonstrated that G at 8 mg/ml was effective against both Gram-positive and Gram-negative bacteria^53^. Similarly, Tian *et al.* (2022) reported that mature biofilm inhibition increased significantly by GA, reaching 74.45% after 24 h treatment using 5 mg/ml^55^. On the other hand, Minich *et al.* (2022) mentioned that S/3-AP/PES and V/3-AP/PES exhibited potential inhibition of bacterial growth and biofilm formation. Both V and S reduced biofilm mass by up to 80% and inhibited EPS production by approximately 55%, particularly against *Staphylococcus epidermidis*. Additionally, both compounds interfered with quorum sensing (QS) signals. They were reported to reduce QS-related gene expression by up to five-fold at sub-inhibitory concentrations (1/20 MIC), downregulating mRNA expression of QS system genes that regulate virulence factor production and motility^4^.

**Fig. 10:**
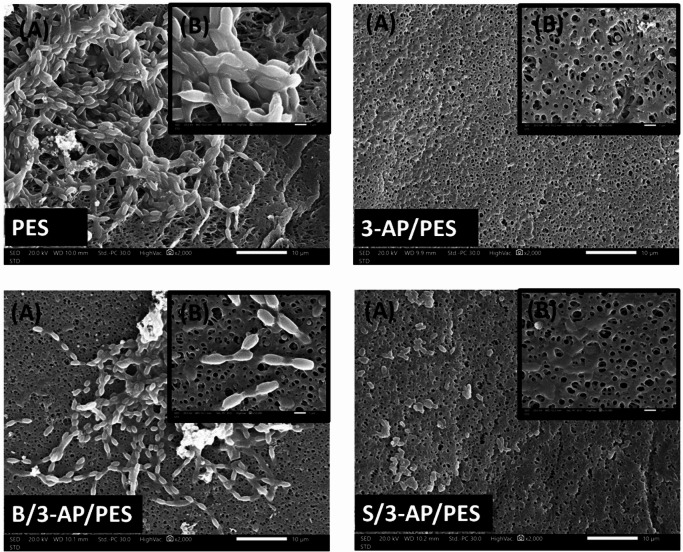
SEM images of PES, 3-AP/PES, B/3-AP/PES, and S/3-AP/PES membranes at (**A**) 2000×, scale bar = 10 µm and (**B**) 10,000× magnification powers, scale bar = 1 μm.

The Scanning Electron Microscopy (SEM) imaging provided direct visual evidence of the biofouling mitigation capacity of the modified membranes. On the unmodified PES membrane, a dense mucilage matrix, secreted by the bacterial community, was clearly observed (Fig. 10), which acts as a protective layer that facilitates biofilm formation. In contrast, this mucilage layer was absent on the one-layered 3-AP/PES membrane. Thin, grafted poly(3-AP) layer formed within a short reaction time (30 min) may impede biofilm formation through steric hindrance and surface hydrophilization, and that hypothesis was previously supported by our team^56^. The S/3-AP/PES membranes exhibited reduced bacterial adhesion compared to the unmodified PES, but retained visible mucilage secretion and impeded bacterial cells, suggesting a distinct decrease in attached bacteria and EPS production. Conversely, the B/3-AP/PES membranes demonstrated an antifouling performance, characterized by bacterial colonization reduction and suppression of mucilage formation. This enhanced performance was reported by our team earlier to be attributed to the brush-like polymeric architecture formed by 3-AP, which increases the effective thickness of the grafted layer, thereby amplifying steric repulsion forces^8,56^.

Some discrepancies arose between SEM observations and TPC results: despite a higher bacterial load detected in TPC assays, SEM images revealed fewer surface-adhered bacteria. This phenomenon highlights the nature of repelling rather than killing especially for the result of the S/3-AP/PES modification. The antimicrobial role of S has been evidenced as a previous study reported its capacity to reduce biofilm and EPS synthesis in *Staphylococcus* by downregulating QS gene expression^4^. The S has been reported to interfere with microbial growth through multiple pathways, including disruption of membrane integrity, alteration of intracellular pH, inhibition of ATP production, and disturbance of proton fluxes. Moreover, recent work has shown that syringic acid synergizes with chitosan to almost completely suppress *E. coli* growth, validating its potential as an effective antibacterial modifier^57^.

### Physical characterization of the dual-layer modified membranes

#### Grafting yield (GY)

Following the second modification step, distinct changes in the coloration of both the modification solutions and the membranes were observed, confirming the occurrence of the grafting reaction. This change intensified with increasing reaction time and substrate concentration. Only minimal color loss occurred during the washing step, which strongly indicates the formation of a covalently bonded grafted layer rather than a loosely adsorbed deposit as mentioned before^33^. The corresponding GY further validated the formation of a new surface layer composed of oligomeric or polymeric structures. At a modifier concentration of 15 mM and a reaction duration of 30 min, the measured GY values were 12.6 µg/cm² for both B/3-AP/PES and S/3-AP/PES, 18.87 µg/cm² for V/3-AP/PES (the highest yield), and 9.44 µg/cm² for G/3-AP/PES (the lowest GY) (Table 2**)**. The comparatively reduced weight of G/3-AP/PES is consistent with earlier reports, which demonstrated the formation of a pancake-like three-dimensional architecture that is substantially thinner than the brush-like structure observed in poly(B)^6^. In contrast, the poly(S) layer exhibited a comparable thickness to that of poly(B).

**Table 2:** Grafting yield by weight difference of modified PES membranes.

Membrane code	Grafting Yield (µg/cm^2^) by weight difference	
	One-layered modification (3-AP/PES)	Dual-Layered modification
B/3-AP/PES	4.72	12.58
G/3-AP/PES		9.44
S/3-AP/PES		12.60
V/3-AP/PES		18.87

#### Membrane hydrophilicity

**Fig. 11:**
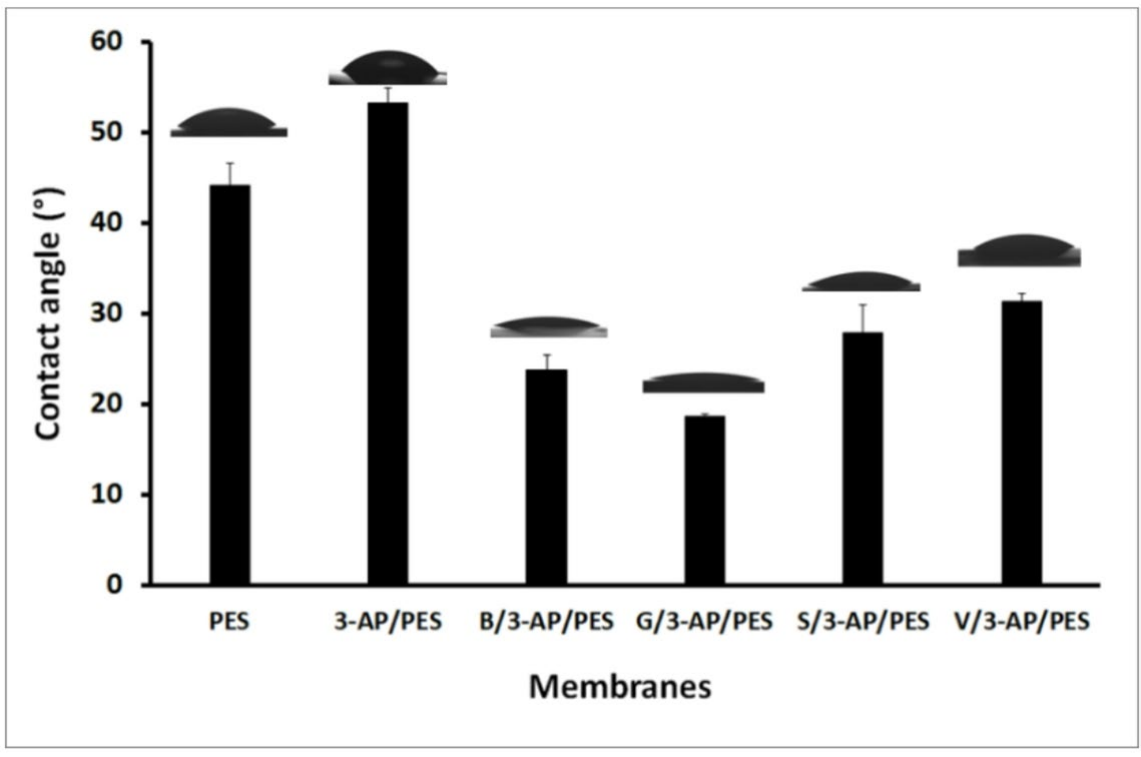
Static water contact angle of unmodified (PES), one-layer modified membrane (3-AP/PES) and dual-layer modified membranes (B/3-AP/PES, G/3-AP/PES, S/3-AP/PES, and V/3-AP/PES).

Static water contact angle (WCA) has indicated enhanced hydrophilicity of the dual-layer modified membranes^58^. At a monomer concentration of 15 mM, B/3-AP/PES and G/3-AP/PES exhibited the lowest WCAs of 23.77° ± 1.52° and 17.87° ± 1.29°, respectively, compared to 44.23° ± 2.38° for the unmodified PES and 53.00° ± 1.79° for one-layered 3-AP/PES (Fig. 11**)**. Although the 3-AP/PES membrane displayed a slightly higher WCA than PES, this effect could be attributed to hydrophilic additives in commercial PES, as pure PES layers on SiO₂ slide typically reach ~ 76°^45^. Nevertheless, 3-AP modification reduced the WCA by 44% relative to PES, consistent with previous reports^8^. Similarly, modifications with B and G yielded lower WCAs than PES, as reported by van der Veen *et al.* (2015)^59^. Hydrophilicity plays a crucial role in determining a membrane’s fouling behavior, as increased surface wettability can significantly reduce bacterial adhesion by repelling hydrophobic microorganisms. Furthermore, the surface roughness of the membrane contributes to this dynamic; while some degree of roughness can enhance surface area and promote hydrophilicity, excessive roughness may inadvertently provide niches for biofilm formation, potentially exacerbating fouling. To elucidate the dominant factors influencing antifouling efficacy, we try to explore the interplay between these characteristics in greater detail. This will include a comprehensive examination of how optimized hydrophilicity and controlled roughness can synergistically enhance membrane performance by minimizing bacterial attachment and biofouling. Ultimately, these insights will facilitate a deeper understanding of the underlying mechanisms in this study^27^.

#### Fourier transform infrared-attenuated total reflection (FTIR-ATR) analysis

**Fig. 12:**
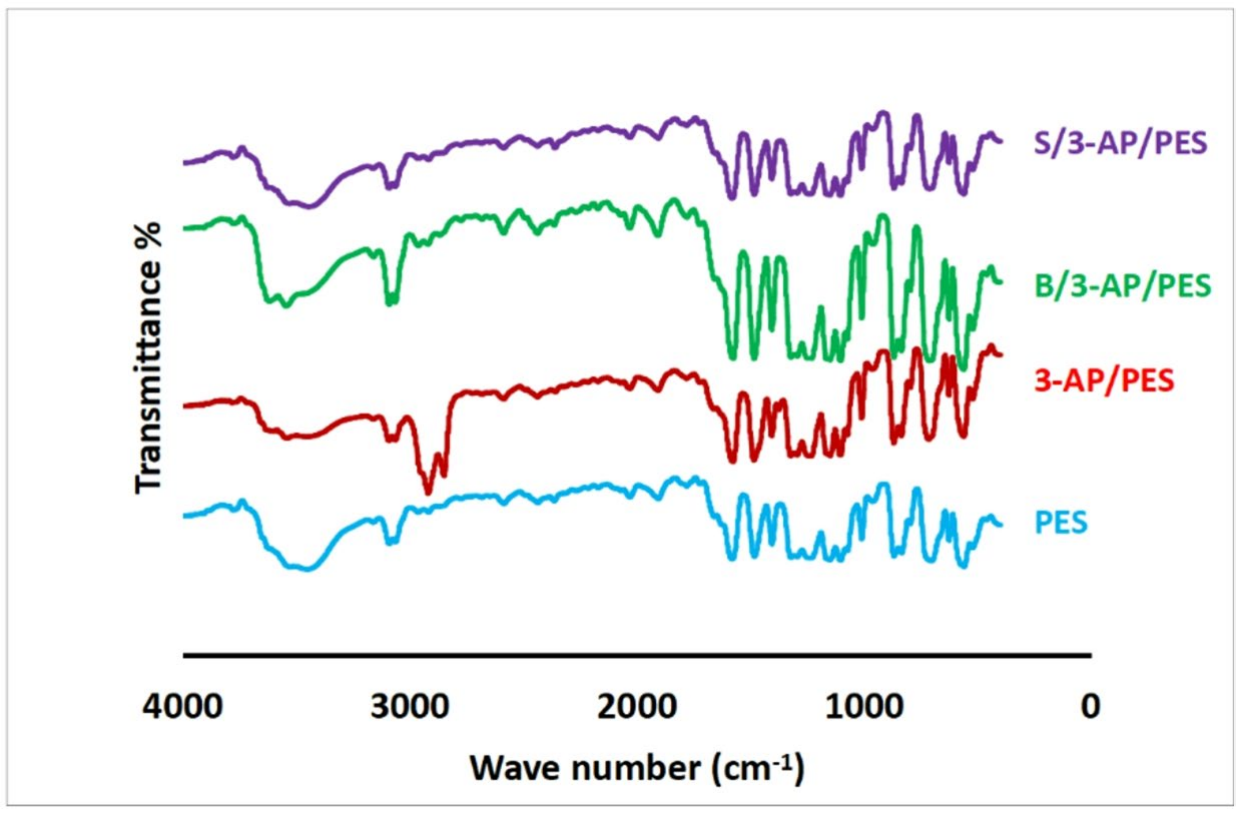
FTIR spectra of the used commercial polyethersulfone (PES) membrane, one-layered 3-AP/PES, and dual-layer modified membranes; B/3-AP/PES, and S/3-AP/PES.

The FTIR spectra of unmodified PES, 3-AP/PES, and dual-layered membranes (B/3-AP/PES and S/3-AP/PES) exhibited characteristic absorption bands confirming the surface modification (Table 3). In PES, peaks at 1569–1401 cm⁻¹ correspond to aromatic C = C stretching, 1348–1113 cm⁻¹ to sulfone (S = O) groups, and 1259 cm⁻¹ to ether (C–O–C) stretching, with a broad band at 3300–3600 cm⁻¹ reflecting phenolic acid additive^12^, as shown in Fig. 12. Upon modification with 3-AP, additional C–H stretching bands appeared at 2921 and 2851 cm⁻¹, while a broad band at 3211–3600 cm⁻¹ indicated N–H stretching, consistent with poly(3-aminophenol) deposition^58^. Dual-layer incorporation of B and S introduced distinctive O–H stretching (3500–3560 cm⁻¹) and bending peaks (1183 cm⁻¹), with increased intensity in the B/3-AP/PES sample. Notably, a reduction in C–H stretching intensities (2921 and 2851 cm⁻¹) in S/3-AP/PES suggests steric hindrance, altered hydrogen bonding, or partial consumption of aliphatic groups during the modification process. Several factors can explain the diminishing intensity of the peaks at 2921 and 2851 cm^−1^: (1) the introduction of phenolic acids may lead to new hydrogen bonding or interactions that alter the vibrational modes of the existing C–H bonds; (2) the added phenolic acid may induce steric hindrance, modifying the conformation of aliphatic chains and reducing the accessibility of C–H groups to the IR beam; or (3) a chemical reaction may has occurred, reducing the number of available C–H bonds in the modified structure. Overall, the FTIR-ATR data confirm the covalent integration of aminophenol and phenolic acids onto PES surfaces, with phenolic incorporation significantly altering hydrogen bonding interactions and structural dynamics of the modified membranes.

**Table 3:** Peak of FTIR-ATR spectrum for PES and modified membranes.

Band position (cm⁻¹)	Functional group assignment	Interpretation	Refs.
1569–1401	Aromatic C = C stretching (PES backbone)	Characteristic PES peaks	^12^
1348–1113	Sulfone (S = O) stretching	Confirms sulfone structure of PES	
1259	Ether (C–O–C) stretching	Confirms ether linkage of PES	
2921, 2851	C–H stretching (symmetric/asymmetric)	Confirms deposition of poly(3-AP) layer, aliphatic chain vibration	^58^
3300–3600	O–H stretching (hydrophilic additives)	Commercial additives enhancing hydrophilicity	
3500	N–H stretching (amine)	Structural feature of poly(3-AP) layer	^60^
3500–3560	O–H stretching (phenolic)	Phenolic acid addition	^12^

#### X-ray diffraction (XRD) analysis

**Fig. 13:**
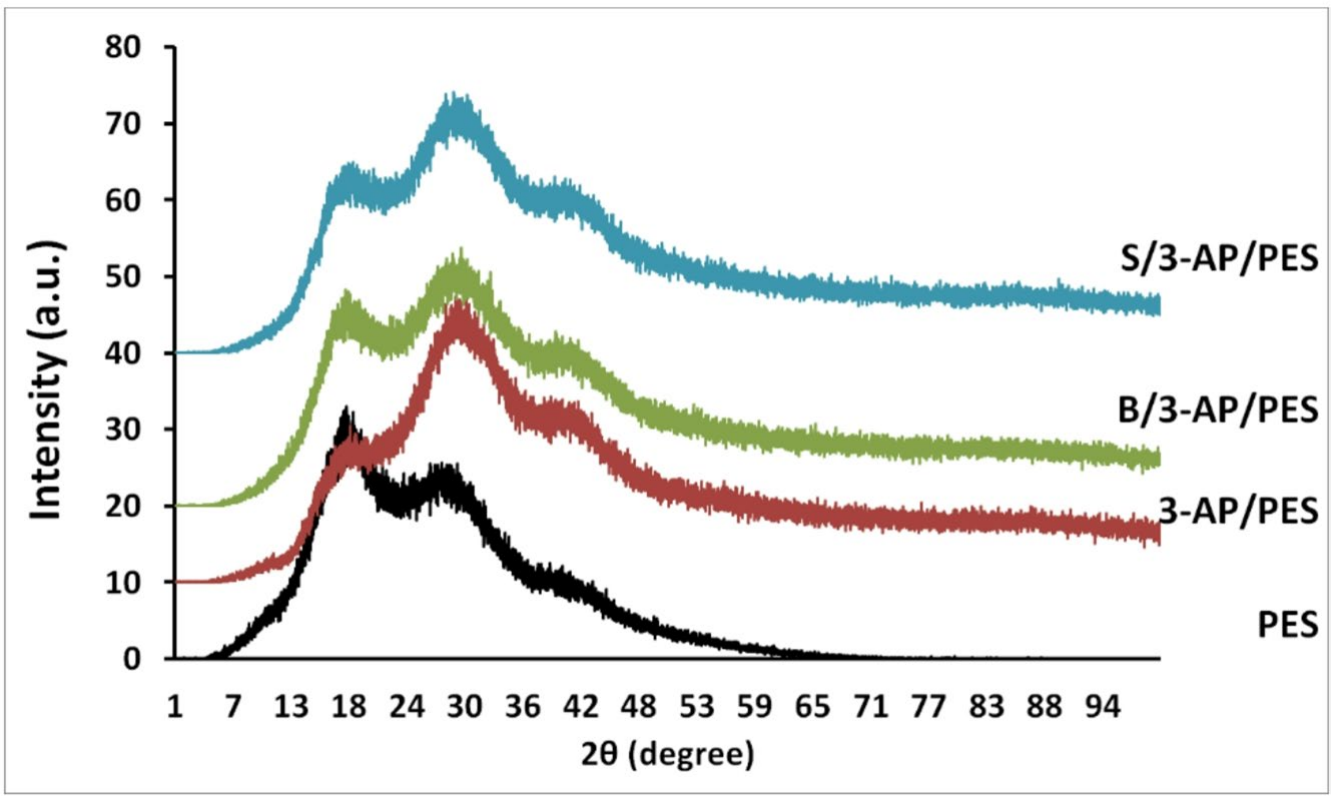
XRD analysis for the unmodified membrane (PES), the modified membranes: 3-AP/PES, B/3-AP/PES, and S/3-AP/PES.

As illustrated in Fig. 13, the XRD spectrum of the unmodified PES membrane exhibits a pronounced broad peak centered at approximately 2θ = 18°, which is consistent with previous reports^61^. In the dual-layer modified membranes (B/3-AP/PES and S/3-AP/PES), additional characteristic peaks appear at 17.5°, 31°, and 42°, indicating structural alterations induced by the grafted phenolic layers. For the one-layer modified membrane (3-AP/PES), the diffraction peak at ~ 17° displayed markedly lower intensity compared to the dual-layered membranes, whereas the peak at 31° exhibited significantly higher intensity. This suggests that the 31° reflection may correspond to a distinct structural feature enhanced during the initial 3-AP modification, possibly reflecting a specific interaction or conformational arrangement of 3-AP within the polymeric layer. Furthermore, the characteristic XRD reflections associated with B and S were reported earlier at 17.6°, 24.5°, and 30°^6^. The ~ 17° peak is attributable to the benzene ring, which is common to both the PES backbone and the phenolic acid structures. In contrast, the reflections at 24.5° and 30° are unique to the phenolic acids, with their intensities increasing proportionally to the extent of grafting onto the PES surface.

#### Scanning electron microscope (SEM) imaging

**Fig. 14:**
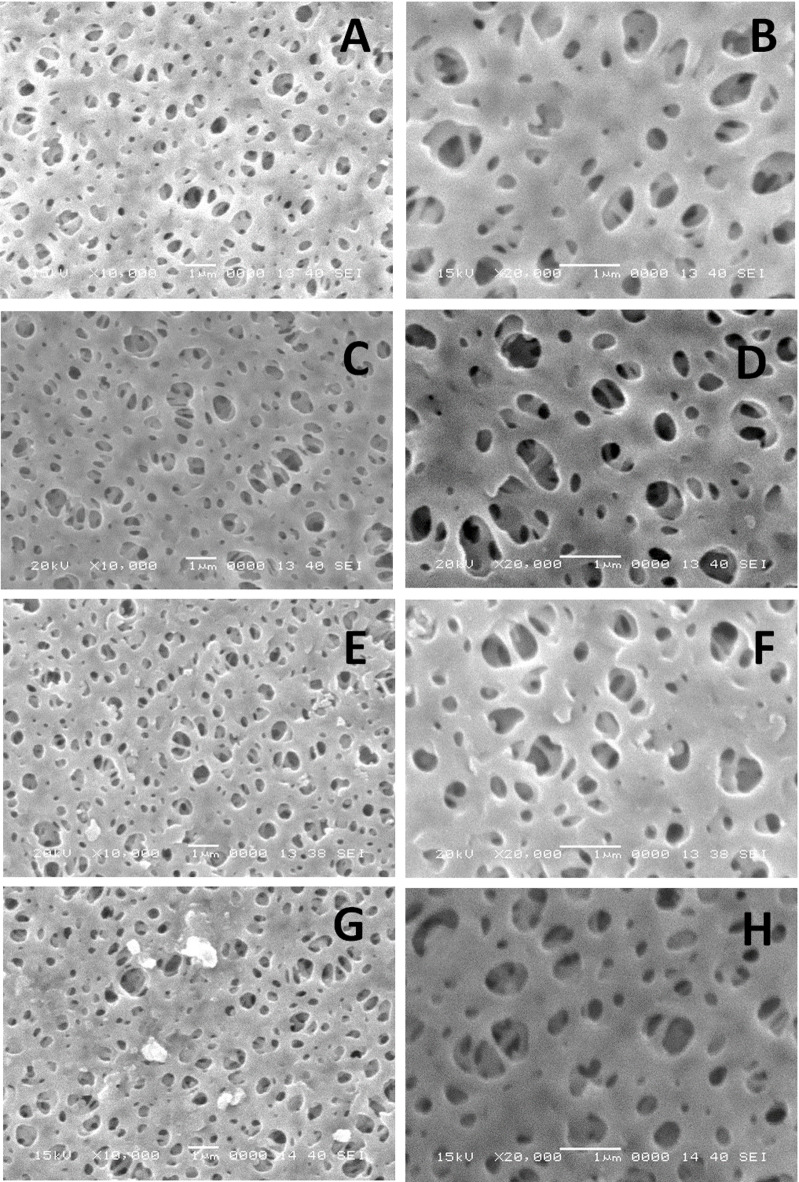
SEM images of the top-views of (**A & B**) PES unmodified membrane surface, (**C & D**) one-layer modified membrane; 3-AP/PES, dual-layer modified membranes; (**E & F**) B/3-AP/PES and (**G & H**) S/3-AP/PES (10,000× and 20,000× magnification with 1 μm scale bar).

SEM imaging was executed to investigate the morphological modification of PES membranes. Micrographs of the unmodified PES membrane, the one-layer modified membrane (3-AP/PES), and the dual-layer modified membranes incorporating 4-hydroxybenzoic acid (B/3-AP/PES) or syringic acid (S/3-AP/PES) are shown in Fig. 14. The SEM images of B/3-AP/PES and S/3-AP/PES revealed a minimal presence of aggregates, primarily composed of homopolymers. These aggregates were substantially less pronounced than those reported in previous studies following prolonged modification (> 2 h) with B^6^, where higher substrate concentrations and extended reaction times promoted aggregate formation^45^. In contrast, the 3-AP/PES membrane exhibited no detectable homopolymer aggregates, which can be attributed to the rapid and uniform initiation of 3-AP polymerization across the membrane surface under the applied conditions^8^.

Surface morphology further indicated an increase in both pore density and pore diameter for B/3-AP/PES and S/3-AP/PES membranes (Fig. 14D and E). This enhancement is likely a result of the introduction of hydrophilic phenolic functional groups, which improve surface wettability as reported by Aljanabi *et al.* (2022)^58^. Similarly, the 3-AP/PES membrane exhibited enlarged, well-preserved open pores (Fig. 14C and F). Additionally, the emergence of new internal cavities was observed, a feature reported to contribute to improved water flux in the modified membranes^45^.

The SEM images indicate a notable pore widening post membrane modification, which raises important considerations regarding the relationship between pore size, surface roughness, and membrane selectivity. An increase in pore size can enhance permeability but may compromise selectivity, allowing a higher flux of undesired molecules alongside target species. In contrast, smaller pores typically improve selectivity by restricting the passage of larger molecules. Additionally, alterations in surface roughness during membrane modification can influence the effective pore size and the interaction dynamics between the membrane surface and permeating molecules. A rougher surface may create additional pathways for flow, subsequently affecting both permeability and selectivity. It is essential to understand the interdependence of these parameters, as an increase in pore size coupled with surface roughness can lead to enhanced flux, potentially at the expense of selective retention. Therefore, optimizing the balance between pore size and surface roughness is crucial for improving membrane performance in various applications. Future design strategies should consider these interactions to achieve desired filtration characteristics effectively.

The pore size analysis detailed in Table 4 provides valuable insights into the structural modifications of unmodified PES and its various modified counterparts. The unmodified PES exhibits a mean pore size of 0.37 μm, serving as a baseline for comparison. The 3-AP/PES membrane shows a notable increase in mean pore size to 0.43 μm, suggesting that the modifications made to this membrane may enhance its permeability. This increase, however, comes with important implications for selectivity, as larger pore sizes can allow a broader range of molecules, including undesired species, to pass through. In contrast, the B/3-AP/PES and S/3-AP/PES membranes exhibit mean pore sizes of 0.32 μm and 0.33 μm, respectively, which are lower than that of the 3-AP/PES membrane. This reduction in pore size could enhance selectivity by limiting the passage of larger or unwanted molecules, thereby improving the filtration performance of these membranes. Notably, the minimum pore sizes across all membranes are relatively close, with the unmodified PES showing the largest minimum pore size of 0.15 μm and the modified membranes ranging slightly lower, reflecting a consistent level of pore restriction. The maximum pore sizes corroborate these trends, with the 3-AP/PES showing the highest maximum pore size at 0.87 μm, which aligns with its mean pore size increase and indicates a potential compromise on selectivity. Meanwhile, maximum pore sizes for B/3-AP/PES and S/3-AP/PES are 0.58 μm and 0.73 μm, respectively, revealing that while these modifications retain some level of permeability, they also focus on preserving selectivity through controlled pore dynamics.

The analysis illustrates a trade-off between permeability and selectivity among the various membranes. The increase in mean pore size in 3-AP/PES suggests enhanced flux, which must be carefully weighed against the risk of reduced selectivity, while the modified the B/3-AP/PES and S/3-AP/PES membranes maintain a focus on selectivity without compromising too much on permeability. These findings highlight the importance of tailoring membrane structures to achieve desired filtration characteristics in various applications.

**Table 4:** The pore size analysis of the unmodified PES and modified membranes.

Membrane code	pore size mean (μm)	Minimum pore size (μm)	Maximum pore size (μm)
PES	0.37 ± 0.13	0.15	0.74
3-AP/PES	0.43± 0.19	0.12	0.87
B/3-AP/PES	0.32 ± 0.11	0.14	0.58
S/3-AP/PES	0.33 ± 0.14	0.14	0.73

#### Atomic force microscope (AFM) imaging

**Fig. 15:**
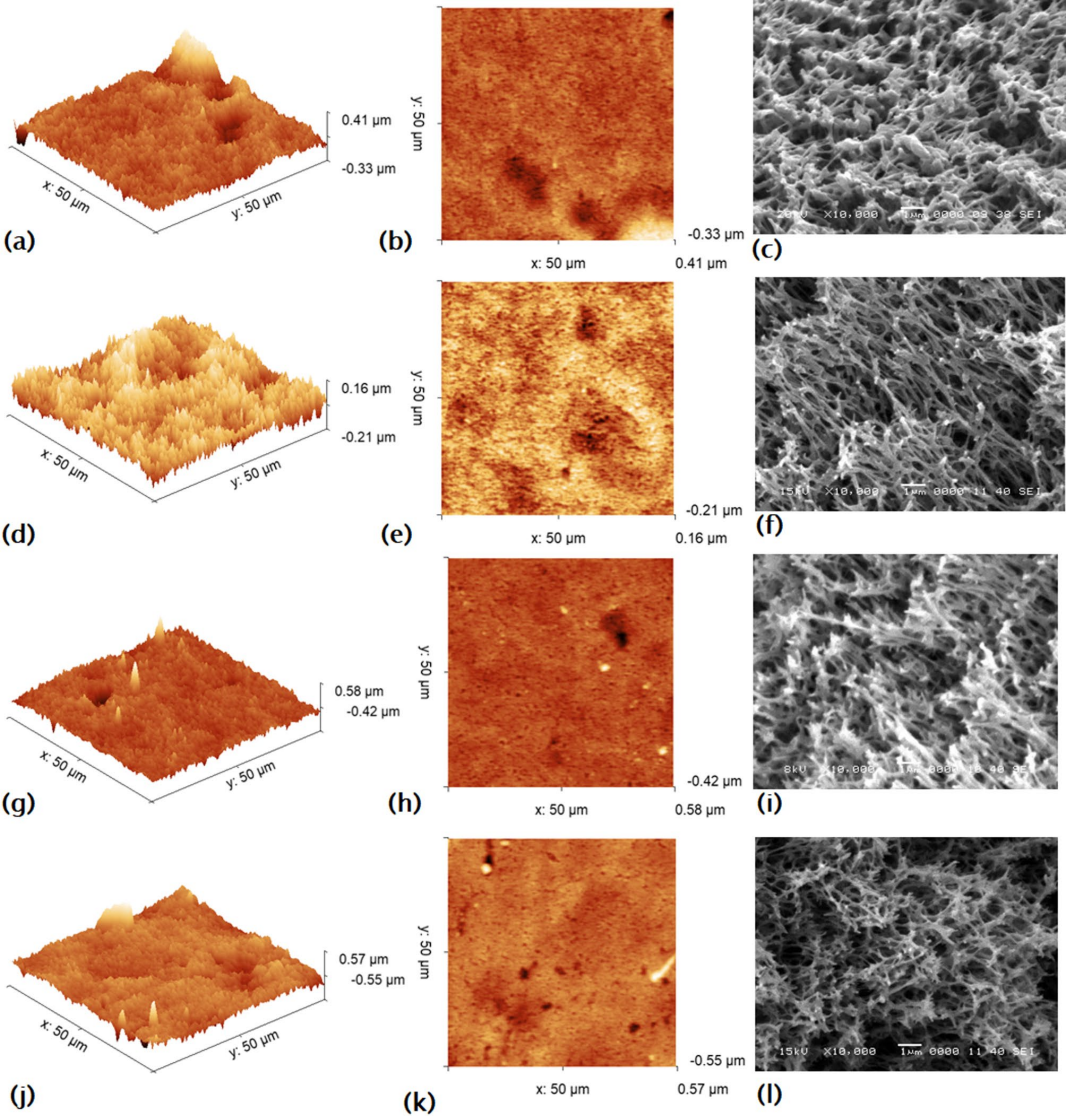
AFM (3D and 2D) images of a 50 × 50 μm area, along with SEM cross-section images, for: (**a–c**) the unmodified PES membrane; (**d–f**) the single-layer modified 3-AP/PES membrane; (**g–i**) the dual-layer modified B/3-AP/PES membrane; and (**j–l**) the S/3-AP/PES membrane (10,000× magnification,1 μm scale bar).

Atomic Force Microscopy (AFM) imaging shown in Fig. 15, confirmed that the modifiers 3-aminophenol (3-AP), 4-hydroxybenzoic acid (B), and syringic acid (S) are capable of independently coupling to the PES surface, leading to the formation of grafted oligomers or polymers, as reported in earlier studies^6,8^. Also, AFM analysis (Fig. 15d) revealed that the 3-AP/PES membrane exhibited a brush-like morphology, which is attributed to the polymerization of 3-AP. Similarly, the B/3-AP/PES membrane (Fig. 15g) demonstrated the formation of linear polymeric chains resembling brush structures, consistent with previous findings^6^. In contrast, the S/3-AP/PES membrane (Fig. 15j) displayed a hybrid morphology, consisting of minor brush-like features combined with a predominant pancake-like layer structure. This observation is further corroborated by SEM cross-sectional images of S/3-AP/PES (Fig. 15j and l), which show the development of such layer accompanied by extended porous margins, aligning with the results reported earlier by Nady *et al.* (2012b)^6^. Additionally, Wang *et al*. (2025) demonstrated that laccase-catalyzed polymerization of syringic acid (S) produces a predominantly pancake-like layer, attributed to the presence of multiple phenolic hydroxyl groups that are capable of generating free radicals, which drive polymerization^57^.

All analytical techniques confirmed that both B and S were effectively and homogeneously grafted onto the PES surface. The modified membranes exhibited a significant increase in surface roughness compared to unmodified PES, as summarized in Table 5. The root-mean-square (RMS) roughness increased from 59.97 nm in the unmodified PES to 183.21 nm in the one-layer 3-AP/PES membrane. The dual-layer B/3-AP/PES membrane displayed a comparable roughness value (180.02 nm) to that of the 3-AP/PES membrane, whereas the dual-layer S/3-AP/PES membrane exhibited a higher roughness (385.13 nm), nearly two-fold greater than B/3-AP/PES.

The observed increase in RMS roughness from 59 nm for the unmodified PES membrane to 385 nm for the S/3-AP/PES membrane presents a counterintuitive scenario, as such a dramatic rise typically correlates with increased fouling. However, in this case, it coincides with enhanced antifouling performance. This phenomenon can be attributed to the distinctive “pancake” morphology exhibited by the modified membranes, which enhances hydrophilicity and reduces the effective contact points for bacterial adhesion. The increased surface area associated with the rougher texture may create spatial advantages that inhibit biofilm formation. Furthermore, the specific interactions between the functional groups of the aminophenol compounds and the waterborne pollutants likely contribute to the observed antifouling effects. These modifications not only alter the physical texture of the membrane but also improve its chemical properties, ultimately leading to superior resistance against fouling agents. This multifaceted interaction underscores the importance of membrane morphology in influencing antifouling performance and necessitates a deeper investigation into the underlying mechanisms. Some researchers referred to the membrane’s surface roughness as a biofilm formation factor, as it increases the area available for bacterial attachment. While other studies suggested that membrane roughness combined with hydrophobicity reduces biofouling by disrupting bacterial adherence and enhances self-cleaning properties through the “lotus effect” as mentioned briefly in our last study^32^.

Relying solely on RMS roughness may yield misleading interpretations, as surface roughness is inherently scale-dependent. Therefore, SAD was also evaluated as a complementary parameter. SAD quantifies the percentage difference between the actual measured surface area and that of a perfectly flat plane with identical x–y dimensions. Both RMS and SAD values were obtained from multiple scans over a 50 × 50 μm area. Unexpectedly, the 3-AP/PES membrane exhibited a lower SAD than the unmodified PES (Table 5), likely due to alterations in pore density and size during modification. Specifically, while the enlargement of pore size could increase RMS roughness, a simultaneous reduction in pore number would counteract this effect, resulting in a lower overall surface area. By contrast, the incorporation of a second modification layer (B/3-AP/PES and S/3-AP/PES) led to a consistent increase in SAD relative to both unmodified PES and the one-layered 3-AP/PES membrane, indicating that dual-layer grafting enhances the effective surface area.

**Table 5:** The root-mean-square (RMS) roughness and the surface area difference (SAD) of the unmodified PES and the modified membranes.

Membrane Code	RMS roughness (nm)	(surface area difference) SAD (%)
PES 3-AP/PES B/3-AP/PES S/3-AP/PES	59.9747 183.211 180.022 385.133	1.176 0.989 1.498 1.858

**Fig. 16:**
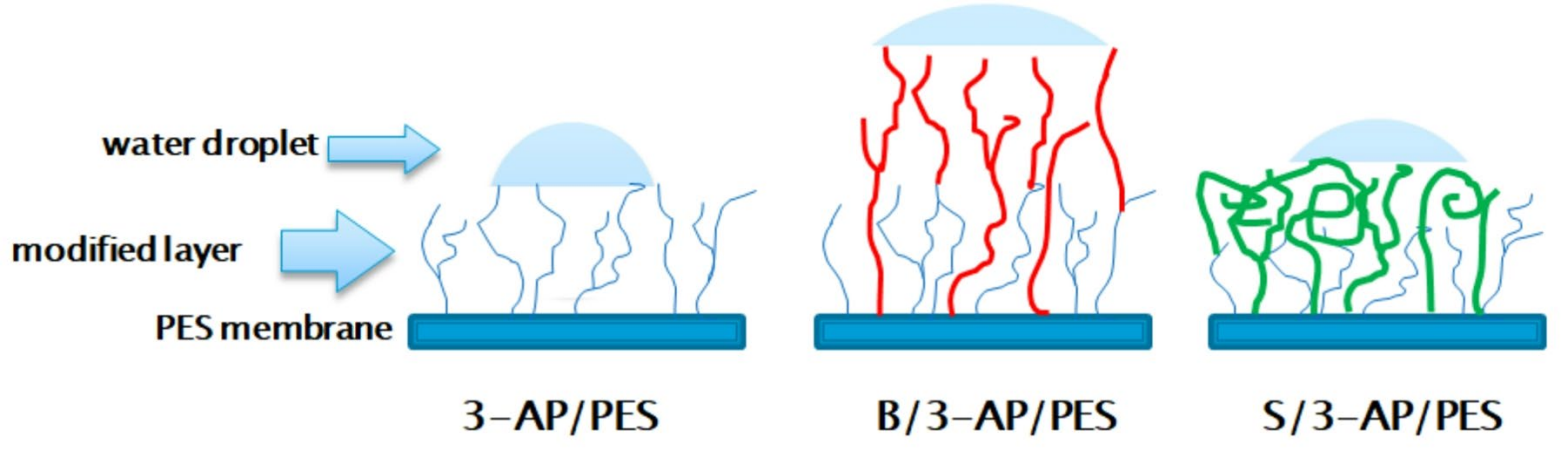
Schematic representation of surface architectures and wettability of one-layer modified PES membranes with 3-aminophenol and dual-layer modified membranes of 4-hydroxybenzoic acid (B/3-AP/PES), and syringic acid (S/3-AP/PES).

Mechanistically, the dual-layered modification may proceed via two complementary pathways: (i) coordination interactions between the amine and hydroxyl groups of 3-AP and the oxygen atoms of 4-hydroxybenzoic acid (B), and (ii) hydrogen bonding and π–π stacking between phenolic hydroxyl groups and the PES surface. Despite the limited cross-linking capacity of 4-hydroxybenzoic acid (B) due to a single hydroxyl substituent, cross-linking via carbon atoms, chain branching, and chain collapse enhances the density and structural integrity of the modified surface^6^. This dual-layered modification strategy effectively integrates 3-aminophenol and B onto the PES membrane surface, thereby enhancing its functional properties. Additionally, syringic acid forms a primarily pancake-like modification layer, as previously mentioned^57^, due to its tendency to create a dense layer of rolled-up branches, as illustrated in Fig. 16. Table 6 presents a comprehensive analysis of various membrane types, focusing on their performance in bacterial and biofilm mitigation, along with their physicochemical properties.

**Table 6:** The most vital parameters of the unmodified PES, one-layer and dual-layer modified membranes.

Experiment analysis	Test	Membranes			
		Blank (PES)	3-AP/PES	B/3-AP/PES	S/3-AP/PES
Bacterial and biofilm mitigation	OD_600_	**-**	**+** **-** **+** **+ +**	**-** **+** **-** **- -**	**- -** **-** **- -** **-**
	TPC	**- -**			
	Hemocytometer	**- - -**			
	SEM	**- - -**			
Water Contact Angle (WCA)		44.1	53. 25°	23.80°	27.85°
AFM	Modifying 2nd layer shape		Brush-like	Brush-like	Pancake-like
	RMS roughness (nm)	59	183	180	385
Grafting Yield (GY)			4.72	12.58	12.60

*(+) is the bacterial inhibiting degree of the membrane and (-) is the bacterial growth enrichment degree of the membrane.

In other effective studies focused on biofouling mitigation, the roughness of iron oxide nanoparticles modified polyacrylonitrile membranes^62^ and polypyrrole modified polysulfone membranes^63^ exhibited reductions of 46% and 97.5%, respectively. To facilitate comparison, Table 7 presents a summary of this study alongside three different anti-biofouling modified membranes, highlighting aspects such as modification techniques, improvements in antifouling capabilities, antibacterial behavior, and overall membrane performance.

The recent study highlights a fascinating interplay between surface roughness and antifouling performance in modified PES membranes. Unlike many previous research efforts that directly associate increased roughness with heightened fouling—typically due to the larger surface area promoting bacterial adhesion—this study reveals that the S/3-AP/PES membrane’s significant increase in RMS roughness from 59 nm to 180 nm is linked to improved antifouling properties. This counterintuitive finding can be attributed to the unique “brush-like” morphology that enhances hydrophilicity, effectively decreasing the available contact points for bacterial attachment. While prior studies have suggested that roughness combined with hydrophobic traits diminishes biofouling, the present research emphasizes that the specific microstructural changes and chemical interactions induced by dual-layer modifications are critical to the superior antifouling behavior observed. This underscores the complexity of membrane design, where a nuanced understanding of morphological characteristics, rather than roughness alone, is essential for optimizing antifouling efficiency.

**Table 7:** A comparative table between our study and three different anti-biofouling modified membranes.

Feature	This study	Ref^62^.	Ref^64^.	Ref^63^.
Modification Technique and Material	Dual-layer Bio-Grafting of aminophenols and phenolic acids onto PES flat sheet membrane using laccase.	MMM using iron oxide nanoparticles in a PAN flat sheet membrane	Chitosan-coated iron oxide nanoparticles in PAN hollow fiber membrane	Blending of polypyrrole (PPy) with polysulfone hollow fiber membrane
Microorganism Types	*Acinetobacter* sp., *Bacillus* sp., *P. stutzeri*, *E. coli*, *S. aureus*	*E. coli*	*P. aeruginosa*,* S. aureus*	*E. coli*,* S. aureus*
Microbial Evaluations	99.9% inhibition; 77% reduction in detached count SEM confirmed the reduction of bacterial adhesion	10% bacterial count reduction with 0.4 wt% Fe_3_O_4_ SEM images showed *E. coli* damage post-adsorption	100% inhibition with 0.4 wt% Fe_3_O_4_ SEM showed *E. coli* cellular integrity loss	70% antibacterial efficiency for *E. coli*; significant biofilm reduction SEM showed significant biofilm reduction
Roughness (RMS) Change	205% increases (Increased to 180.2 nm (B/3-AP/PES membrane)	46% reduction (decreased to 127 nm)	72% reduction (AFM; decreased to 17 nm)	97.5% reduction
Membrane Performance/Antifouling	Contact angle reduced to 23.8°	Porosity decreased to 48%	Permeability increased by 31%; contact angle reduced to 51°	Permeability increased by 31%; contact angle reduced to 52°

## Conclusions

This study uniquely demonstrates the innovative combination of aminophenol and phenolic acids through laccase-catalyzed processes on polyethersulfone (PES) surfaces, a methodology that has not been previously explored in existing dual-layer or bio-inspired coatings, such as polydopamine/phenolic systems. The performance of the dual-layered membranes, specifically the 3-AP/PES and B/3-AP/PES variants, was rigorously evaluated against a bacterial mixture. The 3-AP/PES membrane demonstrated a remarkable 72% reduction in bacterial density (OD_600_) of detached biofilm and a 96% decrease in cell counts, encompassing both viable and non-viable populations, as measured by hemocytometry. Furthermore, the B/3-AP/PES membrane achieved the highest suppression of viable cell attachment, exhibiting a 77% reduction confirmed through total plate count (TPC) analysis. This enhanced antibacterial efficacy is attributed to the bactericidal effect of 4-hydroxybenzoic acid (B), which significantly increased the count of non-viable bacteria. Optimal bacterial repellence was achieved at 25 and 35 °C, pH 7.5, and salinities of 35 and 57 ppt. Contact angle measurements demonstrated enhanced hydrophilicity of the dual-layer membranes, with B/3-AP/PES (23.77°) and G/3-AP/PES (17.87°) exhibiting the lowest contact angle values at a monomer concentration of 15 mM, corresponding to 46% and 60% reduction compared to the unmodified membrane, respectively. SEM analyses revealed thicker, stacked internal fibers and new cavities, which may contribute to improved membranes’ water flux especially in B/3-AP/PES and S/3-AP/PES membranes. Microscopic examination and physical analyses verified that B and S were homogeneously grafted onto the PES surface. Structural characterizations indicated that B/3-AP/PES favors linear brush-like chains, while S/3-AP/PES forms predominantly pancake-like layers. The RMS roughness increased substantially from 59.97 nm (unmodified PES) to 183.21 nm (3-AP/PES) and further to 385.13 nm (S/3-AP/PES). Collectively, these results underscore the potential of these modified membranes as effective strategies for combating bacterial biofilm formation.

This study introduces a novel approach to polyethersulfone (PES) membrane modification through the innovative combination of aminophenol and phenolic acids via laccase-catalyzed processes, creating a dual-layer system that significantly enhances biofouling resistance. Notably, the B/3-AP/PES and S/3-AP/PES membranes exhibit improved hydrophilicity and increased roughness, resulting in substantial bacterial inhibition (up to 99.9%) and reduced bacterial adhesion. This innovative strategy not only aims to lower membrane replacement costs but also contributes to environmental sustainability by protecting water bodies from contaminants. The findings underline the potential of this dual-layer modified membrane system in advancing water treatment technologies. In light of the critical need for sustainable solutions in biofilm management, this study elucidates the efficacy of a low-protein adsorption modifying layer engineered to enhance anti-biofouling properties. Through comprehensive investigations, we have demonstrated the layer’s remarkable selectivity and robustness, positioning it as a promising candidate for a variety of applications. However, we recognize that long-term performance metrics, including reusability and stability under prolonged operational conditions, have not yet been fully explored. Acknowledging this gap, we propose to incorporate further studies focused on the durability of the modified membranes, thereby solidifying the foundation for their practical deployment in real-world scenarios.

## Data Availability

All data for this article, including tables and figures, are included in the manuscript.
